# Cytotoxic Polyhydroxysteroidal Glycosides from Starfish *Culcita novaeguineae*

**DOI:** 10.3390/md16030092

**Published:** 2018-03-13

**Authors:** Yunyang Lu, Hu Li, Minchang Wang, Yang Liu, Yingda Feng, Ke Liu, Haifeng Tang

**Affiliations:** 1 Institute of Materia Medica, School of Pharmacy, Fourth Military Medical University, Xi’an 710032, China; luyunyanggq@163.com (Y.L.); pharm_lihu@163.com (H.L.); so870823@163.com (Y.L.); fyd1991@sina.com (Y.F.); 2 First Motorized Detachment of Shanghai Armed Police Corps, Shanghai 200126, China; 3 Nuclear Magnetic Resonance Center, Xi’an Modern Chemistry Research Institute, Xi’an 710065, China; wmc204@163.com (M.W.); happycoco5133@163.com (K.L.)

**Keywords:** *Culcita novaeguineae*, starfish, polyhydroxysteroidal glycoside, cytotoxicity

## Abstract

Four new polyhydroxysteroidal glycosides—culcinosides A–D (**1**, **2**, **4**, and **7**)—along with three known compounds—echinasteroside C (**3**), linckoside F (**5**), and linckoside L3 (**6**)—were isolated from the ethanol extract of starfish *Culcita novaeguineae* collected from the Xisha Islands of the South China Sea. The structures of new compounds were elucidated through extensive spectroscopic studies and chemical evidence, especially two-dimensional (2D) NMR techniques. The cytotoxicity of the new compounds against human glioblastoma cell lines U87, U251, and SHG44 were evaluated.

## 1. Introduction

**Figure 1:**
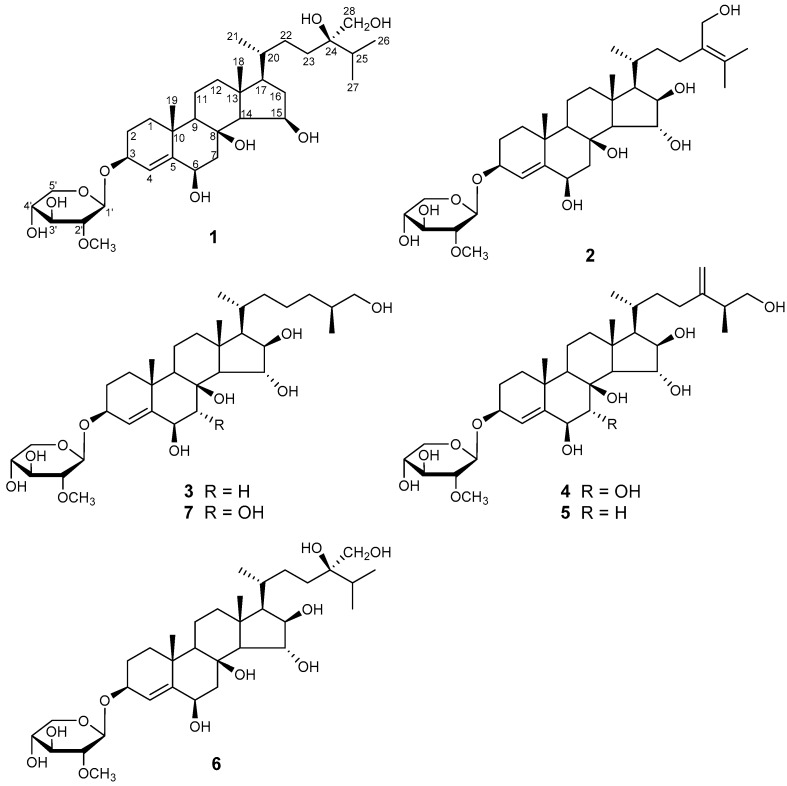
The structures of compounds **1**–**7** isolated from starfish *Culcita novaeguineae*.

The starfish is a sea animal belonging to Asteroidea: Echinodermata that is distributed worldwide; there are approximately 1900 species grouped into 370 genera [1]. Steroidal glycosides are the predominant metabolites of starfish, and are responsible for their general toxicity. Based on structural characteristics, they have been subdivided into three groups: asterosaponins, polyhydroxysteroidal glycosides, and cyclic steroidal glycosides [2]. Polyhydroxysteroidal glycosides are abundant in the metabolism of starfish; more than 500 have been identified in total [3]. In general, polyhydroxysteroidal glycosides consist of an oxygenated steroidal aglycone with more than three hydroxy groups, and one or two (rarely three) monosaccharide residues attached to the steroidal nucleus or side chain. Polyhydroxysteroidal glycosides have been reported to show a broad spectrum of biological activities, including hemolytic, cytotoxic, immunoregulatory, anti-bacterial, neuritogenic, anti-inflammatory, and anti-biofouling effects [4,5,6,7,8,9,10,11,12]. *Culcita novaeguineae* is plentiful in the South China Sea; it is used as a folk medicine for the treatment of rheumatism, and as a tonic in China [13]. The chemical constituent investigation of this starfish has led to the isolation of several polyhydroxysteroid glycosides and asterosaponins by scientists around the world [14,15]. The previous work that our team carried out on *Culcita novaeguineae* led to the isolation of a series of novel asterosaponins. Some of these have shown significant cytotoxicity against several human cancer cell lines, such as asterosaponin 1, through suppressing the proliferation of human glioblastoma cell line U87 with an IC_50_ of 4.3 μg/mL [2,6,16,17,18,19]. However, no polyhydroxysteroidal glycosides, as a large class of bioactive metabolites of starfish, were found in our previous work. Therefore, as part of a continuous search for bioactive steroidal glycosides from starfish, we aimed for the polyhydroxysteroidal glycosides in *Culcita novaeguineae*. Herein, we report the isolation, structural elucidation, and biological activity screening of four new polyhydroxysteroidal glycosides, culcinosides A–D (**1**, **2**, **4***,* and **7**), together with three known compounds, which were identified as echinasteroside C (**3**), linckoside F (**5**), and linckoside L3 (**6**), through a comparison of the physical and spectra data with literature values (Figure 1) [10,12,20].

## 2. Results and Discussion

### 2.1. Structure Elucidation

**Figure 2:**
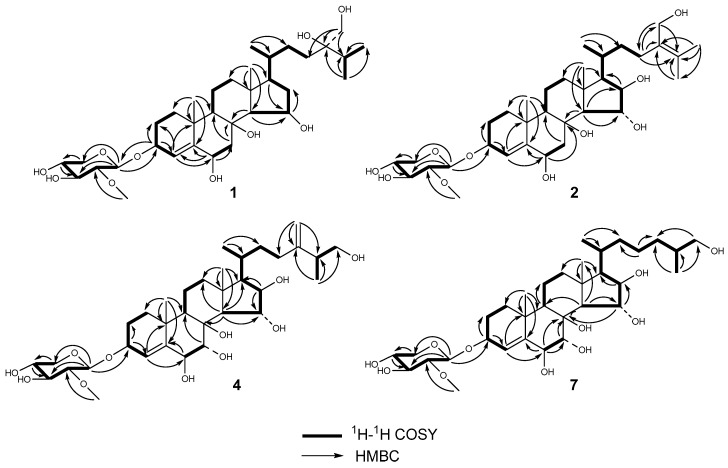
The key ^1^H-^1^H COSY and HMBC correlations of the new compounds culcinosides A–D (**1**, **2**, **4**, and **7**).

**Figure 3:**
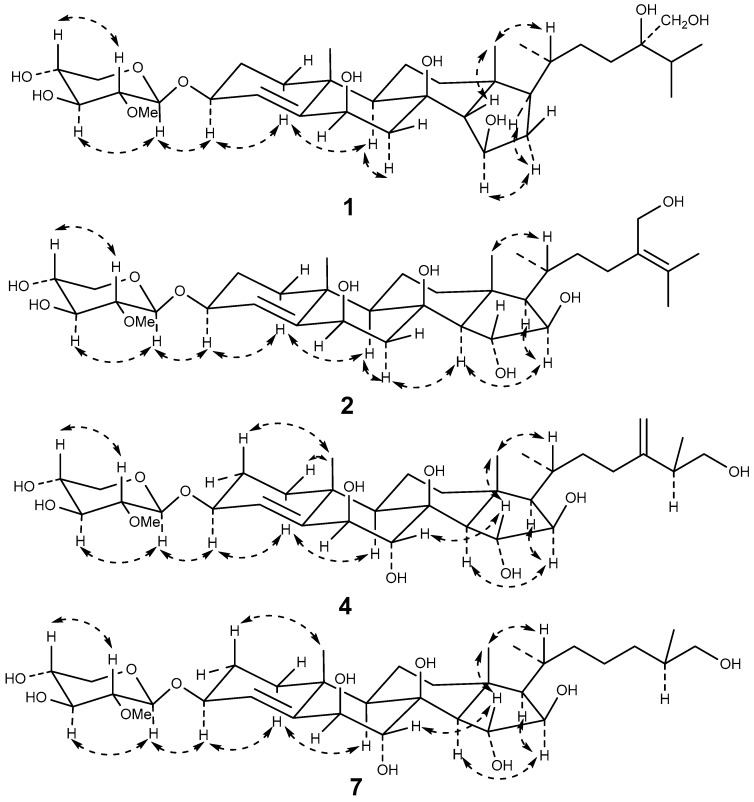
The key NOESY correlations of the new compounds culcinosides A-D (**1**, **2**, **4**, and **7**).

Culcinoside A (**1**) was isolated as a colorless powder, and was positive for the Liebermann–Burchard and Molisch tests, which indicated that it might be a steroidal glycoside. The molecular formula of compound **1** was determined as C_34_H_58_O_10_ by ESIMS at *m*/*z* 649 [M + Na]^+^ and HRESIMS at *m*/*z* 649.3942 [M + Na]^+^ (calculated for C_34_H_58_O_10_Na, 649.3928). The ^1^H-NMR, ^13^C-NMR, and DEPT spectra suggested the existence of a trisubstituted double bond (δ_C_ 126.5, δ_H_ 5.94; δ_C_ 148.9), two angular methyls (δ_C_ 16.0, C-18; δ_C_ 23.1, C-19) with singlets (δ_H_ 1.30, H-18; δ_H_ 1.66, H-19), three doublets (δ_H_ 1.07, δ_C_ 19.4, C-21; δ_H_ 1.22, δ_C_ 18.0, C-26; δ_H_ 1.20, δ_C_ 18.1, C-27), three oxidized methines (δ_C_ 69.5, δ_H_ 4.90; δ_C_ 75.9, δ_H_ 4.77; δ_C_ 76.6, δ_H_ 4.48), and two oxygenated quaternary carbon (δ_C_ 76.0, δ_C_ 76.2). A comprehensive analysis and comparison of the above data with that of co-isolated linckoside L3 (**6**) indicated that they may share the same steroidal aglycone and side chain (Table 1). A difference was recognized in ring D, where a secondary alcohol (δ_C_ 83.1, δ_H_ 4.04) in compound **6** was replaced by one methylene (δ_H_ 2.15 and 2.27, δ_C_ 42.3, CH_2_-16) of compound **1**. Furthermore, this substitution was witnessed by the upfield shift of C-15 (δ_C_ 69.5, δ_H_ 4.90) in compound **1** when compared with that of **6** (δ_C_ 80.6, δ_H_ 4.17). The position of the double bond was determined by HMBC correlations for H-4 to C-2, C-6, and C-10; H-3 and H-5 to C-4 and C-5; and H-19 to C-5. This connection was further confirmed by the ^1^H-^1^H COSY correlations for H-3 to H-4. The assignment of the NMR signals associated with the aglycone moiety and side chain (Table 1) was derived from HSQC, ^1^H-^1^H COSY, HMBC, and TOCSY experiments. The normally occurring 2-*O*-methyl-β-d-xylopyranose (2-OMe-Xyl) moiety in the polyhydroxysteroidal glycosides was detected in compound **1** by analyzing the one-dimensional (1D) and two-dimensional (2D) NMR spectra, and by comparing the monosaccharide signals with those co-isolated known compounds and the literature values [21,22,23]. This was further confirmed by the demethylation and acid hydrolysis of compound **1** with 2 M trifluoroacetic acid, followed by derivatization with 1-(trimethylsily)-imidazole, gas chromatography (GC) analysis sequentially, and a comparison with the corresponding derivatives of a standard monosaccharide [6,8]. The attachment of the monosaccharide to C-3 was confirmed by HMBC correlation for H-3 to C-1′ (δ_C_ 104.8) of the Xyl, and H-1′ (δ_H_ 4.84) of the Xyl to C-3 of the aglycone (Figure 2). The NOESY correlations of H-3 to H-1b, H-1b to H-9, H-6 to H-7b, and H-7b to H-9, and the lack of correlation between H-1 and H-6 indicated the α-orientation of H-3 and H-6 (Figure 3). The β-orientation of the hydroxy at C-15 was determined by the NOESY correlation of H-16b to H-15 and H-17, and the lack of correlation between H-14 and H-15. The 20*R* configuration was deduced from the NOESY correlation of H-20 to H-18, and the large coupling constant of H-17 (*J* = 9.1) [10]. It has been reported that small but important differences in the signals for the HC(28), H’C(28), and C(28) atoms in the NMR spectra of the synthetic 24*R* and 24*S* epimers of 24-hydroxymethyl-24-hydroxycholesterol (24*R*: ∆δ_H_ = 0.04, δ_C_ = 66.0; 24*S*: ∆δ_H_ = 0.06, δ_C_ = 66.3) [12]. The NMR spectroscopic data of compound **1** at CH_2_-28 (∆δ_H_ = 0.04, δ_C_ = 66.1) coincided with that of the 24*R* epimer. Thus, the absolute configuration of C-24 was determined as *R*. Therefore, the structure of compound **1** was established as (24*R*)-3-*O*-(2-*O*-methyl-β-d-xylopyranosyl)-cholesta-4-ene-3β,6β,8,15β,24,28-hexaol.

Culcinoside B (**2**), a colorless powder, was positive for the Liebermann–Burchard and Molisch tests, which indicated that it might be a steroidal glycoside. The molecular formula of compound **2** (C_34_H_56_O_10_) was deduced from HRESIMS at *m*/*z* 647.3795 [M + Na]^+^ (calculated for C_34_H_56_O_10_Na, 647.3771). The ^1^H, ^13^C, and DEPT NMR spectra signals belonging to the tetracyclic moiety of the aglycone of compound **2** revealed the presence of two angular methyls (δ_C_ 17.5, δ_H_ 1.74 s, CH_3_-18; δ_C_ 23.1, δ_H_ 1.67 s, CH_3_-19), one trisubstituted double bond 4(5) (δ_C_ 126.5, δ_H_ 5.94, C-4; δ_C_ 149.0, C-5), three oxygenated methines (δ_C_ 76.0, δ_H_ 4.76, CH-6; δ_C_ 80.8, δ_H_ 5.08, CH-15; δ_C_ 83.0, δ_H_ 4.78, CH-16), one oxygenated methine (CH-3) bearing a monosaccharide residue (δ_C_ 76.7, δ_H_ 4.48), and one quaternary oxygenated carbon C-8 (δ_C_ 76.0). All of the above data were similar to the known compounds echinasteroside C (**3**) and linckoside A, which possessed the same ∆^4^-3β,6β,8,15α,16β-pentahydroxycholestane aglycone [11,20]. The chemical shift of the anomeric proton at δ_H_ 4.84 (*J* = 7.6) associated with the anomeric carbon at δ_C_ 104.8 in the HSQC spectrum and the carbon signals at δ_C_ 61.2, 67.5, 71.6, 78.16, and 85.6 suggested the presence of the 2-*O*-methyl-β-d-xylopyranose monosaccharide residue, and this was further confirmed by acid hydrolysis followed by GC analysis. The connection of the monosaccharide to C-3 was deduced from the HMBC correlation for H-1′ of Xyl to C-3 of the aglycone (Figure 2). All of the H and C signals of compound **2** were assigned by the 2D NMR spectra, including HSQC, ^1^H-^1^H COSY, and HMBC (Table 1). The structure of the side chain for compound **2** was elucidated on the basis of 2D NMR spectra. The tetrasubstituted double bond at C-24 and C-25, and the C-28 hydroxymethyl were determined by HMBC correlations for H-23 (2H) to C-24, C-25 and C-28; H-26 and H-27 to C-24 and C-25; and H-28 (2H) to C-23, C-24, and C-25. The 3β, 6β, 15α, and 16β orientations for the aglycone of **2** were confirmed by the cross-peaks of the NOESY spectrum (Figure 3). The 20*R* configuration was deduced from the NOESY correlation of H-20 to H-18, and Hα-13 to H-21. According to the data above, the structure of compound **2** was elucidated as 3-*O*-(2-*O*-methyl-β-d-xylopyranosyl)-cholesta-4,24-diene-3β,6β,8,15α,16β,28-hexaol.

Culcinoside C (**4**), a colorless powder, was positive for the Liebermann–Burchard and Molisch tests, which indicated that it might be a steroidal glycoside. The HRESIMS at *m*/*z* 663.3696 [M + Na]^+^ (calculated for C_34_H_56_O_1__1_Na, 663.3720) indicated that the molecular formula of **4** was C_34_H_56_O_11_. The ^1^H, ^13^C, and DEPT NMR spectra revealed two double bonds, including one trisubstituted double bond (δ_C_ 130.3, δ_H_ 5.70, C-4; δ_C_ 145.4, C-5) and one terminal double bond (δ_C_ 154.0, C-28; δ_C_ 109.4, δ_H_ 4.77, 4.84), one anomeric carbon (δ_C_ 104.7 with δ_H_ 4.44), two angular methyl (δ_C_ 17.0, δ_H_ 1.16 s, CH_3_-18; δ_C_ 23.2, δ_H_ 1.34 s, CH_3_-19), one hydroxymethyl (δ_C_ 67.6, δ_H_ 3.38, 3.59), five oxygenated methines (δ_C_ 77.5, δ_H_ 4.24, C-3; δ_C_ 79.9, δ_H_ 4.13, C-6; δ_C_ 73.9, δ_H_ 3.98, C-7; δ_C_ 80.1, δ_H_ 4.20, C-15; and δ_C_ 82.6, δ_H_ 4.03, C-16), and one oxygenated quaternary carbon (δ_C_ 78.0, C-8). All of the chemical shifts belonging to compound **4** were similar to those of the known compound linckoside F (**5**) from the starfish *Linckia laevigata* [10]. A detailed comparison of the NMR spectra for compounds **4** and **5** indicated that the only difference was that one methylene (δ_C_ 44.6, δ_H_ 2.04, 3.39, CH_2_-7) in **5** was replaced by an oxygenated methine in **4** (δ_C_ 73.9, δ_H_ 3.98, CH-7). The assignments of the NMR signals associated with compound **4** were derived from the HSQC, ^1^H-^1^H COSY, HMBC, and TOCSY experiments (Table 1). The NOESY correlation for H-7 to H-15 and H-18 to H-15 suggested the α orientation of C-7 (Figure 3). The 20*R* configuration was deduced from the NOESY correlation of H-18 to H-20, and the chemical shift of H-21 δ_H_ 0.96 (δ_H_ 0.90–0.96 for 20*R* steroid). The stereochemistry at C-25 was expected as *S* by analogy with co-occurring compound **5**, and on the basis of the comparison of their NMR spectra. Thus, the structure of **4** was established as (25*S*)-3-*O*-(2-*O*-methyl-β-d-xylopyranosyl)-cholesta-4,24(28)-diene-3β,6β,7α,8,15α,16β,26-heptaol.

Culcinoside D (**7**) was obtained as a colorless powder. The positive results of the Liebermann–Burchard and Molisch tests suggested that it might be a steroidal glycoside. The molecular formula was determined as C_33_H_56_O_11_ by HRESIMS at *m*/*z* 651.3740 [M + Na]^+^ (calculated for C_33_H_56_O_11_Na, 651.3720). The ^1^H-NMR, ^13^C-NMR, and DEPT spectra of compound **7** revealed the presence of a steroidal aglycone with two angular methyls (δ_C_ 17.6, δ_H_ 1.77, CH_3_-18; δ_C_ 23.6, δ_H_ 1.68, CH_3_-19), one 4(5) double bond (δ_C_ 129.8, δ_H_ 6.17, C-4; δ_C_ 146.2, C-5), five oxygenated methine groups (δ_C_ 76.6, δ_H_ 4.51, C-3; δ_C_ 80.0, δ_H_ 4.87, C-6; δ_C_ 74.5, δ_H_ 4.84, C-7; δ_C_ 80.4, δ_H_ 5.11, C-15; δ_C_ 82.5, δ_H_ 4.72, C-16), and a quaternary carbon (δ_C_ 78.1, C-8) bearing a hydroxy group. The anomeric carbon (δ_C_ 104.7) with δ_H_ at 4.76 (d, 7.6) indicated the presence of the common 2-*O*-methyl-β-d-xylopyranosyl moiety, and their ^1^H and ^13^C signals were assigned by the 2D NMR data (Table 1). The only difference between compounds **7** and **3**, which was elucidated as echinasteroside C isolated from the starfish *Echinaster brasiliensis*, was that a methylene (δ_C_ 45.0, δ_H_ 2.10 and 3.45, CH_2_-7) in compound **3** was substituted by one oxygenated methine (δ_C_ 74.5, δ_H_ 4.84, CH-7) in compound **7** [20]. Therefore, the structure of **7** was elucidated as (25*S*)-3-*O*-(2-*O*-methyl-β-d-xylopyranosyl)-cholesta-4-ene-3β,6β,7α,8,15α,16β,26-heptaol.

### 2.2. Cytotoxic Activities

The cytotoxic activity of the new compounds **1**, **2**, **4**, and **7** against human glioblastoma cell lines U87, U251, and SHG44 were evaluated using the 3-(4,5-dimethylthiazol-2-yl)-2,5-diphenyltetrazolium bromide (MTT) colorimetric assay method in vitro [24]. Compound **1** exhibited cytotoxicity against the three cancer cell lines, and compounds **2**, **4**, and **7** showed moderate activity (Table 2). Doxorubicin was used as the positive control.

## 3. Experimental Section

### 3.1. General Experimental Procedures

Optical rotations were measured with a Perkin-Elmer 343 polarimeter (German Perkin-Elmer Corporation, Boelingen, Germany). 1D and 2D NMR spectra were recorded on a Bruker AVANCE III 500 and 800 MHz spectrometer with TMS (Tetramethylsilane) as the internal standard. ESIMS and HRESIMS were carried out on a Micromass Quattro mass spectrometer (Waters, Shanghai, China). HPLC was carried out on a Dionex P680 liquid chromatograph (Dionex, Germering, Germany) equipped with a UV 170 UV/Vis detector using a YMC-Pack C18 column (20 × 250 mm i.d., 5 μm, YMC Co., Ltd., Kyoto, Japan) and monitored at 206 nm, 225 nm, 275 nm, and 300 nm, simultaneously. GC was performed on a Finnigan Voyager apparatus using an l-Chirasil-Val column (25 m × 0.32 mm i.d.) for the analyses of the trimethylsilyated hydrolysates. Column chromatographies were performed on silica gel (200–300 mesh and 300–400 mesh; Qingdao Marine Chemical Inc., Qingdao, China), reversed phase silica gel (Lichroprep RP-18, 40–63 µm, Merck Inc., New York, NY, USA), and Sephadex LH-20 (40–70 µm, GE-Healthcare, Uppsala, Sweden). Chemical reagents for isolation were of analytical grade, and purchased from Tianjin Fuyu Chemical Co. Ltd. (Tianjin, China).

### 3.2. Animal Material

The starfish were collected from the South China Sea (Xisha Islands, Sansha, Hainan Province, China) in August 2015. The organisms were identified as *Culcita novaeguineae* by Dr. Ning Xiao (Institute of Oceanology, Chinese Academy of Science, Qingdao, China). A voucher specimen (No. HX201508) was deposited in the Institute of Materia Medica, School of Pharmacy, Fourth Military Medical University (Xi’an, China).

### 3.3. Extraction and Isolation

The starfish (80.0 kg, wet weight) were cut into pieces, and then extracted with 75% ethanol three times, each time for 2 h under reflux. The extract was combined and dried in vacuo to leave a residue, which was suspended in water and then partitioned with petroleum ether and *n*-butanol sequentially. The *n*-butanol part (320.0 g) was subjected to silica gel column chromatography eluting with a CHCl_3_/CH_3_OH/H_2_O (50:1:0 to 6:3.5:1) gradient to give 16 major fractions, which were obtained based on TLC analysis. Fraction 10 was subjected to size exclusion chromatography on a Sephadex LH-20 column eluting with CHCl_3_/CH_3_OH (1:1) to remove the impurities, then was further purified by HPLC to give compounds **1** (3.4 mg, *t*_R_ = 35.5 min) and **2** (1.6 mg, *t*_R_ = 57.6 min), eluting with CH_3_CN/H_2_O (2:3) at a flow rate of 6 mL/min. RP-C_18_ column chromatography eluted with CH_3_OH:H_2_O (1:1 to 1:0) and Sephadex LH-20 column chromatography equilibrated with CHCl_3_/CH_3_OH (1:1) were used successively on the purification of fraction 11 to obtain the subfraction fr.11-2-4. Finally, fraction 11-2-4 was subjected to HPLC eluting with CH_3_CN/H_2_O (1:1.5) to afford compounds **3** (57.1 mg, *t*_R_ = 25.6 min), **4** (57.2 mg, *t*_R_ = 27.3 min), and **5** (141.3 mg, *t*_R_ = 29.3 min). Fraction 12 (6.1 g) was subjected to size exclusion chromatography on a Sephadex LH-20 column equilibrated with CHCl_3_/CH_3_OH (1:1) to remove impurities and give three subfractions (Fr.12-1 to Fr.12-3). The subfraction 12-2 (2.2 g) was purified by a RP-C_18_ column chromatography eluting with CH_3_OH:H_2_O (3:2 to 1:0) to give 10 subfractions. Then, fraction 12-2-8 (150.0 mg) was purified by semi-preparative HPLC eluting with CH_3_CN/H_2_O (2:3) at a flow rate of 6 mL/min to yield compound **6** (54.0 mg, *t*_R_ = 27.2 min). Compound **7** (26.6 mg, *t*_R_ = 33.0 min) was obtained from fraction 12-2-9 by HPLC eluting with CH_3_CN/H_2_O (2:3) at a flow rate of 6 mL/min.

### 3.4. Spectral and Physicochemical Data of New Compounds

Culcinoside A (**1**): C_34_H_58_O_10_, colorless powder, [α]${}_{D}^{22}$ −13.6 (*c* 0.03, MeOH), ^1^H- and ^13^C-NMR data are shown in Table 1; ESIMS *m*/*z* 649 [M + Na]^+^; HRESIMS *m*/*z* 649.3942 [M + Na]^+^ (calculated for C_34_H_58_O_10_Na, 649.3928).

Culcinoside B (**2**): C_34_H_56_O_10_, colorless powder, [α]${}_{D}^{22}$ −10.3 (*c* 0.04, MeOH), ^1^H- and ^13^C-NMR data are shown in Table 1; ESIMS *m*/*z* 647 [M + Na]^+^; HRESIMS *m*/*z* 647.3795 [M + Na]^+^ (calculated for C_34_H_56_O_10_Na 647.3771).

Culcinoside C (**4**): C_34_H_56_O_11_, colorless powder, [α]${}_{D}^{22}$ −28.4 (*c* 0.21, MeOH), ^1^H- and ^13^C-NMR data are shown in Table 1; ESIMS *m*/*z* 663 [M + Na]^+^; HRESIMS *m*/*z* 663.3696 [M + Na]^+^ (calculated for C_34_H_56_O_1__1_Na 663.3720).

Culcinoside D (**7**): C_33_H_56_O_11_, colorless powder, [α]${}_{D}^{22}$ −16.5 (*c* 0.11, MeOH), ^1^H- and ^13^C-NMR data are shown in Table 1; ESIMS *m*/*z* 651 [M + Na]^+^; HRESIMS *m*/*z* 651.3740 [M + Na]^+^ (calculated for C_33_H_56_O_11_Na, 651.3720).

### 3.5. Demethylation and Acid Hydrolysis of the New Compounds

The new compounds (each 1.5 mg) were mixed with 1 mL of dry dichloromethane and 0.01 mL of boron tribromide at −80 °C for 30 min, and then stood overnight at 10 °C under anhydrous conditions. The solvent and reagent were evaporated to dryness in vacuo at room temperature. The demethylated derivative of the new compound was heated with 1.0 mL of trifluoroacetic acid (TFA) at 120 °C for 2 h. The reaction mixture was evaporated in vacuo, and the residue was partitioned between CH_2_Cl_2_ and H_2_O. The aqueous phase was concentrated and dissolved in 1-(trimethylsilyl)imidazole and anhydrous pyridine (0.1 mL). Then, the solution was stirred at 60 °C for 5 min, and dried with a stream of N_2_. The residue was partitioned between CH_2_Cl_2_ and H_2_O. The CH_2_Cl_2_ layer was analyzed by GC with an initial temperature of 100 °C for 1 min, and then temperature programmed to 180 °C at a rate of 5 °C/min. The peak of the derivative of the sample was detected at 11.25 and 12.46 min for compound **1**, 11.25 and 12.45 min for compound **2**, 11.23 and 12.45 for compound **4**, and 11.24 and 12.26 min for compound **7**. The retention time of the authentic samples after being treated simultaneously with 1-(trimethylsily)imidazole in pyridine were 11.23 and 12.44 min (d-xylose), and 11.34 and 12.40 min (l-xylose), respectively [6].

### 3.6. Assays for In Vitro Cytotoxicity

The cytotoxicity of new compounds **1**, **2**, **4**, and **7** against human glioblastoma cell lines U87, U251, and SHG44 were evaluated by the 3-(4,5-dimethylthiazol-2-yl)-2,5-diphenyltetrazolium bromide (MTT) colorimetric assay method in vitro. All of the cells were cultured in RPMI-1640 medium supplemented with 10% fetal bovine serum, 100 U/mL benzyl penicillin, and 100 U/mL streptomycin at 37 °C in a humidified atmosphere with 5% CO_2_. The logarithmic phase cells were seeded on 96-well plates at a concentration of 4 × 10^3^ cell/mL, and incubated with various concentrations (100 μM, 80 μM, 60 μM, 40 μM, 20 μM, 10 μM, 1 μM, and 0.25 μM in medium containing less than 0.1% DMSO) of test compounds in triple wells for 48 h, and doxorubicin was used as the positive control. Next, 20 μL MTT (5 mg/mL) was added to each well, and incubated for another 4 h. The water-insoluble dark blue formazan crystals formed during MTT cleavage in actively metabolizing cells were dissolved in DMSO. The optical density of each well was measured with a Bio-Rad 680 microplate reader at 570 nm. Cytotoxicity was expressed as the concentration of drug inhibiting cell growth by 50% (IC_50_).

## Figures and Tables

**Table 1:** The ^1^H-NMR and ^13^C-NMR data of compounds **1**, **2**, **4**, and **7** (δ in ppm, *J* in Hz).

Position	1 ^c,e^		2 ^d,e^		4 ^c,f^		7 ^c,e^	
	δ_C_	δ_H_	δ_C_	δ_H_	δ_C_	δ_H_	δ_C_	δ_H_
1a	39.4	1.85 m	39.4	1.86 m	39.6	1.77 m	39.4	1.90 m
1b		1.39 m		1.40 m		1.32 m		1.57 m
2a	28.2	2.28 m	28.2	2.28 m	28	1.99 m	28.3	2.28 m
2b		2.09 m		2.09 m		1.76 m		2.12 m
3	76.6	4.48 brt (7.2)	76.7	4.48 m	77.5	4.24 brt (8.2)	76.6	4.51 m
4	126.5	5.94 s	126.5	5.94 s	130.3	5.70 s	129.8	6.17 s
5	148.9	-	149	-	145.4	-	146.2	-
6	75.9	4.77 d (2.5)	76	4.76 d (2.3)	79.9	4.12 d(3.1)	80	4.87 d(2.7)
7a	44.9	3.40 dd (14.8, 2.5)	44.9	3.49 dd (14.7, 2.3)	73.9	3.96 d(3.1)	74.5	4.84 d(2.7)
7b		2.07 m		2.08 m				
8	76	-	76	-	78	-	78.1	-
9	57.6	1.24 m	60.8	1.55 m	51.5	1.34 m	51.3	1.91 m
10	37.6	-	37.7	-	37.4	-	37.3	-
11a	19.7	2.10 m	19.6	2.15 m	19.4	1.89 m	19.5	2.27 m
11b		1.55 m		1.57 m		1.53 m		1.67 m
12a	42.5	2.07 m	43	2.14 m	43.1	1.98 m	43	2.16 m
12b		1.28 m		1.32 m		1.22 m		1.40 m
13	45	-	45	-	45.5	-	45.3	-
14	66.6	1.56 m	64.1	1.51 m	59.5	1.42 d (10.6)	59.7	2.05 d (10.5)
15	69.5	4.90 m	80.8	5.08 m	80.1	4.20 dd (10.6, 1.8)	80.4	5.11 brd (10.5)
16a	42.3	2.27 m	83	4.78 m	82.6	4.03 dd (7.4, 1.8)	82.5	4.72 dd (7.1, 1.2)
16b		2.15 m						
17	55.8	1.61 brd (9.1)	60.8	1.55 m	61.4	1.29 dd (10.8, 7.4)	61.6	1.54 m
18	16	1.30 s	17.5	1.74 s	17	1.16 s	17.6	1.77 s
19	23.1	1.66 s	23.1	1.67 s	23.2	1.34 s	23.6	1.68 s
20	36.6	1.54 m	30.6	2.42 m	30.7	1.90 m	30.5	2.40 m
21	19.4	1.07 d (6.2)	19.1	1.21 d (6.7)	18.5	0.96 d (6.7)	18.8	1.14 d (6.7)
22a	30.6	1.92 m	35.7	2.20 m	35.4	1.74 m	37.1	1.96 m
22b		1.41 m		1.54 m		1.23 m		1.33 m
23a	32	2.03 m	29.2	2.55 t (7.3)	33	2.15 m	25	1.69 m
23b		1.85 m		1.52 m		1.97 m		1.44 m
24a	76.2	-	135	-	154	-	35	1.64 m
24b								1.23 m
25	34	2.26 m	128.1	-	43.5	2.31 m	37.3	1.82 m
26a	18	1.20 d (6.8)	20.7	1.75 s	67.6	3.58 m	68	3.76 dd (10.4, 5.6)
26b						3.37 m		3.64 m
27	18.1	1.22 d (6.8)	21.1	1.77 s	17.4	1.07 d (6.5)	18	1.08 d (6.7)
28a	66.1	4.03 m	62.4	4.51 m	109.4	4.84 s	-	-
28b		3.97 m		4.42 m		4.77 s		
2-OMe-Xyl								
1′	104.8	4.84 d (7.6)	104.8	4.84 d (7.6)	104.7	4.44 d (7.6)	104.7	4.76 d (7.6)
2′	85.6	3.46 dd (8.8, 7.6)	85.6	3.46 dd (8.8, 7.6)	85	2.85 dd (9.0, 7.6)	85.5	3.44 dd (8.9, 7.6)
3′	78.2	4.08 m	78.2	4.08 br.t(8.8)	77.6	3.33 m	78.1	4.04 br.t (8.9)
4′	71.6	4.23 m	71.6	4.23 m	71.4	3.50 m	71.6	4.20 m
5′a	67.5	4.35 dd (11.3, 5.4) 3.67 m	67.5	4.34 dd (11.4, 5.4)	66.9	3.84 dd (11.5, 5.4)	67.5	4.32 dd (11.3, 5.3)
5′b				3.67 t (11.4)		3.18dd (11.5, 10.4)		3.62 m
2-OMe	61.2	3.72 s	61.2	3.72 s	61.3	3.59 s	61.1	3.68 s

^c^ The NMR data were recorded at 500 MHz for δ_H_ and 125 MHz for δ_C_; ^d^ the NMR data were recorded at 800 MHz for δ_H_ and 200 MHz for δ_C_; ^e^ in C_5_D_5_N; ^f^ in CD_3_OD.

**Table 2:** Cytotoxic activity of the new compounds in vitro (mean ± SD, *n* = 3).

Compounds	Cytotoxic Activity (IC_50_, µM)		
	U87	U251	SHG44
**1**	9.35 ± 0.46	11.28 ± 0.65	8.04 ± 0.32
**2**	33.52 ± 1.23	40.76 ± 1.58	36.54 ± 1.44
**4**	26.33 ± 1.16	22.66 ± 1.28	35.26 ± 1.51
**7**	43.25 ± 1.73	28.93 ± 1.83	26.22 ± 1.64
Doxorubicin	0.33 ± 0.02	0.24 ± 0.01	0.15 ± 0.01

## Supplementary Materials

The NMR and HRESIMS data of the new compounds and the ^13^C-NMR data of the known compounds are available online at http://www.mdpi.com/1660-3397/16/3/92/s1.

## Abbreviations

The following abbreviations are used in this paper:

DEPT	Distortionless enhancement by polarization transfer
ESIMS	Electron spray ionization mass spectrum
^1^H-^1^H COSY	^1^H-^1^H Correlation spectroscopy
HMBC	^1^H-detected heteronuclear multiple bond correlation
HPLC	High performance liquid chromatography
HRESIMS	High resolution electron spray ionization mass spectrum
HSQC	^1^H-detected heteronuclear single quantum correlation
NMR	Nuclear magnetic resonance
NOESY	Nuclear overhauser enhancement spectroscopy
TLC	Thin layer chromatography
TOCSY	Total correlation spectroscopy
