# Supplementary material for: Cytotoxic Polyhydroxysteroidal Glycosides from Starfish Culcita novaeguineae
Source: Mar Drugs. 2018 Mar 13;16(3):92. doi: 10.3390/md16030092 (PMC5867636; doi:10.3390/md16030092)
Supplement: Supplementary file 1 [file marinedrugs-16-00092-s001.pdf]

Cytotoxic Polyhydroxysteroidal Glycosides from Starfish *Culcita novaeguineae*

Yunyang Lu[1], Hu Li[1,2], Minchang Wang[3], Yang Liu[1], Yingda Feng[1], Ke Liu[3] and Haifeng Tang [1,]*

[1] Institute of Materia Medica, School of Pharmacy, Fourth Military Medical University, Xi'an 710032, China

[2] First Motorized Detachment of Shanghai Armed Police Corps, Shanghai 200126, China

[3] Nuclear Magnetic Resonance Center, Xi'an Modern Chemistry Research Institute, Xi'an 710065, China

***** Correspondence: tanghaifeng71@163.com; Tel.: +86-29-8477-4748

Table S1. The ^{13}C NMR data of compounds echinasteroside C (**3**), linckoside F (**5**) and linckoside L3 (**6**).

Table S2. The inhibition ratio of the new compounds against glioblastoma cell lines at 24h.

Table S3. The inhibition ratio of the new compounds against glioblastoma cell lines at 48h.

Table S4. The inhibition ratio of the new compounds against glioblastoma cell lines at 72h.

Figure S1. ^{1}H NMR (500 MHz, in C_5D_5N) spectrum of Culcinoside A (**1**)

Figure S2. ^{13}C NMR (125 MHz, in C_5D_5N) spectrum of Culcinoside A (**1**)

Figure S3. HSQC spectrum of Culcinoside A (**1**)

Figure S4. HMBC spectrum of Culcinoside A (**1**)

Figure S5. ^{1}H-^{1}H COSY spectrum of Culcinoside A (**1**)

Figure S6. NOESY spectrum of Culcinoside A (**1**)

Figure S7. ^{1}H NMR (800 MHz, in C_5D_5N) spectrum of Culcinoside B (**2**)

Figure S8. ^{13}C NMR (200 MHz, in C_5D_5N) spectrum of Culcinoside B (**2**)

Figure S9. HSQC spectrum of Culcinoside B (**2**)

Figure S10. HMBC spectrum of Culcinoside B (**2**)

Figure S11. ^{1}H-^{1}H COSY spectrum of Culcinoside B (**2**)

Figure S12. ^{1}H NMR (500 MHz, in CD_3OD) spectrum of Culcinoside C (**4**)

Figure S13. ^{13}C NMR (125 MHz, in CD_3OD) spectrum of Culcinoside C (**4**)

Figure S14. HSQC spectrum of Culcinoside C (**4**)

Figure S15. HMBC spectrum of Culcinoside C (**4**)

Figure S16. ^{1}H-^{1}H COSY spectrum of Culcinoside C (**4**)

Figure S17. NOESY spectrum of Culcinoside C (**4**)

Figure S18. ^{1}H NMR (500 MHz, in C_5D_5N) spectrum of Culcinoside D (**7**)

Figure S19. ^{13}C NMR (125 MHz, in C_5D_5N) spectrum of Culcinoside D (**7**)

Figure S20. HSQC spectrum of Culcinoside D (**7**)

Figure S21. HMBC spectrum of Culcinoside D (**7**)

Figure S22. ^{1}H-^{1}H COSY spectrum of Culcinoside D (**7**)

Figure S23. NOESY spectrum of Culcinoside D (**7**)

Figure S24.HRESIMS spectrum of Culcinoside A (**1**)

Figure S25. HRESIMS spectrum of Culcinoside B (**2**)

Figure S26.HRESIMS spectrum of Culcinoside C (**4**)

Figure S27.HRESIMS spectrum of Culcinoside D (**7**)

Table S1. The ^{13}C-NMR (500 MHz) data of compounds echinasteroside C (**3**), linckoside F (**5**) and linckoside L3 (**6**) (*δ* in ppm).

position	3[a]	5[a]	6[b]
1	39.5	39.1	39.8
2	28.3	28.0	28.0
3	76.8	76.4	77.6
4	126.6	126.2	127.0
5	149.5	148.6	148.6
6	76.1	75.7	76.5
7	45.0	44.6	44.5
8	76.1	75.7	76.3
9	57.8	57.4	57.9
10	37.8	37.4	37.8
11	19.7	19.3	19.6
12	43.1	42.7	43.1
13	45.2	44.8	45.2
14	64.3	63.8	63.7
15	81.3	80.9	80.6
16	85.6	82.5	83.1
17	60.9	60.4	61.1
18	17.7	17.3	16.9
19	23.2	22.8	22.9
20	30.7	30.2	31.8
21	19.1	18.6	18.8
22	37.3	35.0	30.2
23	25.1	32.8	31.7
24	35.1	153.9	77.5
25	37.3	43.4	33.9
26	68.1	67.0	17.3

27	18.1	17.5	17.6
28		108.9	66.2
2-OMe-Xyl			
1'	104.9	104.5	104.7
2'	85.6	85.2	85.0
3'	78.1	77.7	77.6
4'	71.7	71.3	71.3
5'	67.5	67.1	66.9
2-OMe	61.3	60.9	61.3

[a] in C_5D_5N. [b] in CD_3OD.

Table S2. The inhibition ratio of the new compounds against glioblastoma cell lines at 24h.

Compounds	Inhibition ratio (%)		
	U87	U251	SHG44
1	19.32	17.71	21.27
2	10.16	8.47	9.33
4	12.14	13.35	9.82
7	7.78	11.22	11.88

The concentration of each compound was 10 μmol/L.

Table S3. The inhibition ratio of the new compounds against glioblastoma cell lines at 48h.

Compounds	Inhibition ratio (%)		
	U87	U251	SHG44
1	54.85	48.63	55.68
2	19.91	16.44	18.75
4	26.26	28.45	19.42
7	15.81	25.57	26.14

The concentration of each compound was 10 μmol/L.

Table S4. The inhibition ratio of the new compounds against glioblastoma cell lines at 72h.

Compounds	Inhibition ratio (%)		
	U87	U251	SHG44
1	63.42	61.38	65.88
2	25.73	21.82	22.95
4	34.26	37.13	24.35
7	20.64	33.58	34.62

The concentration of each compound was 10 μmol/L.

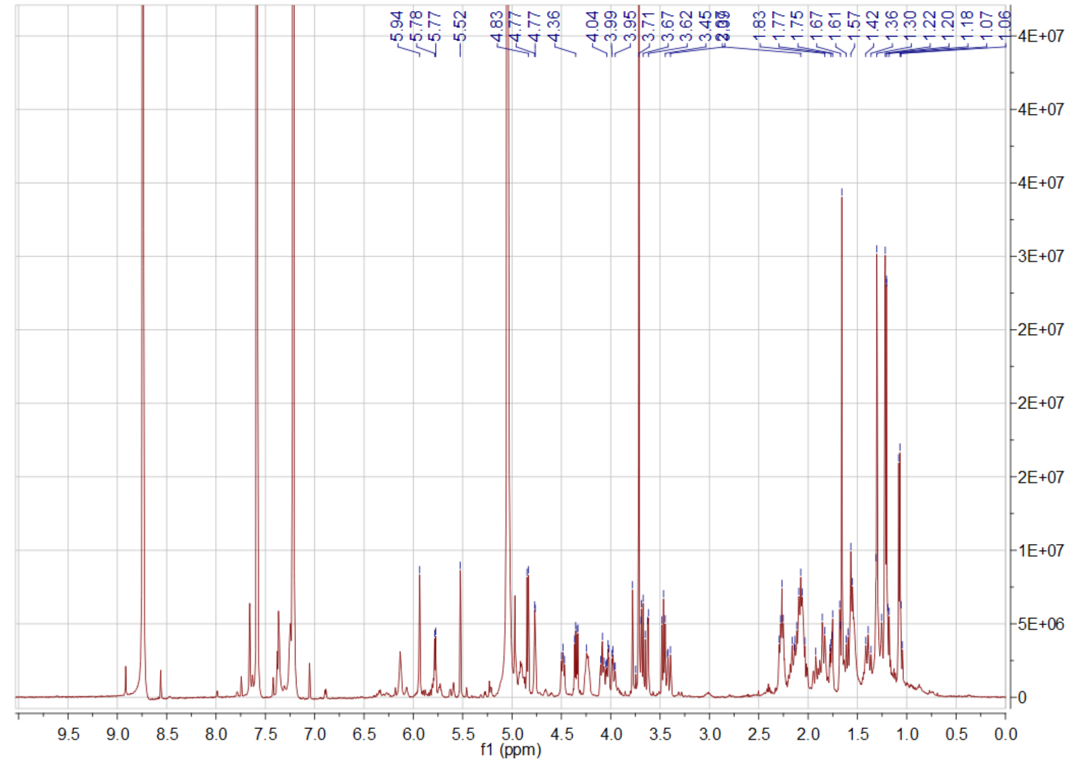

Figure S1. ^{1}H NMR (500 MHz, in C_5D_5N) spectrum of Culcinoside A (**1**)

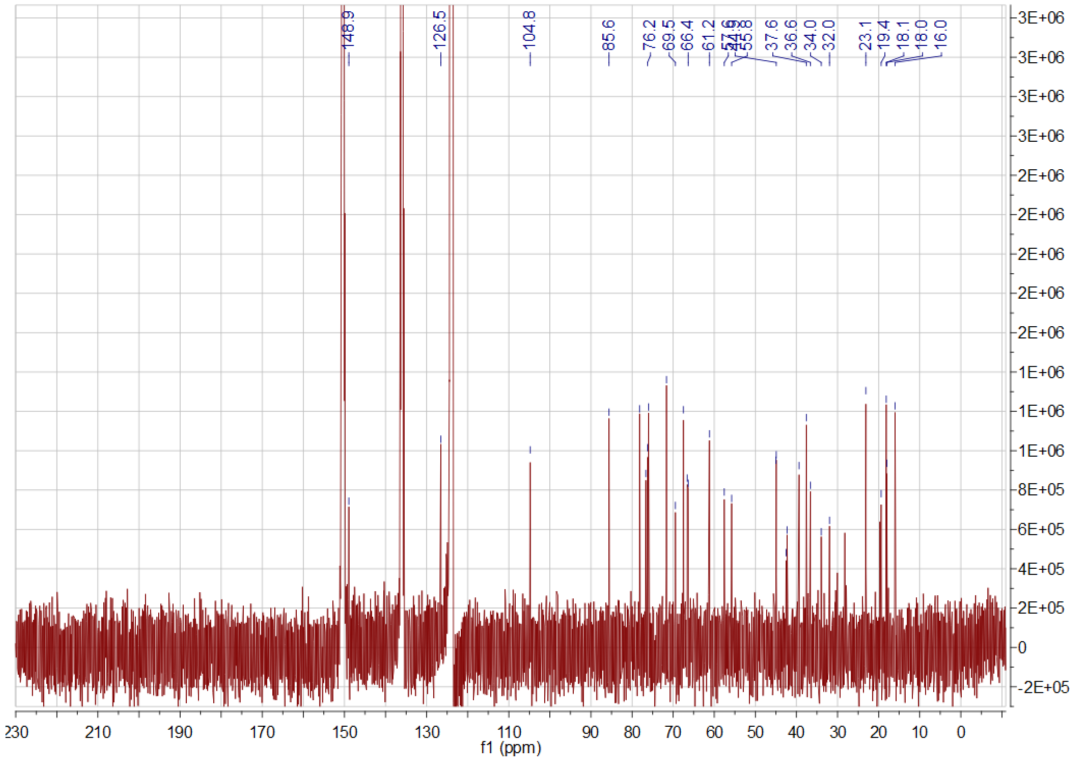

Figure S2. ^{13}C NMR (125 MHz, in C_5D_5N) spectrum of Culcinoside A (**1**)

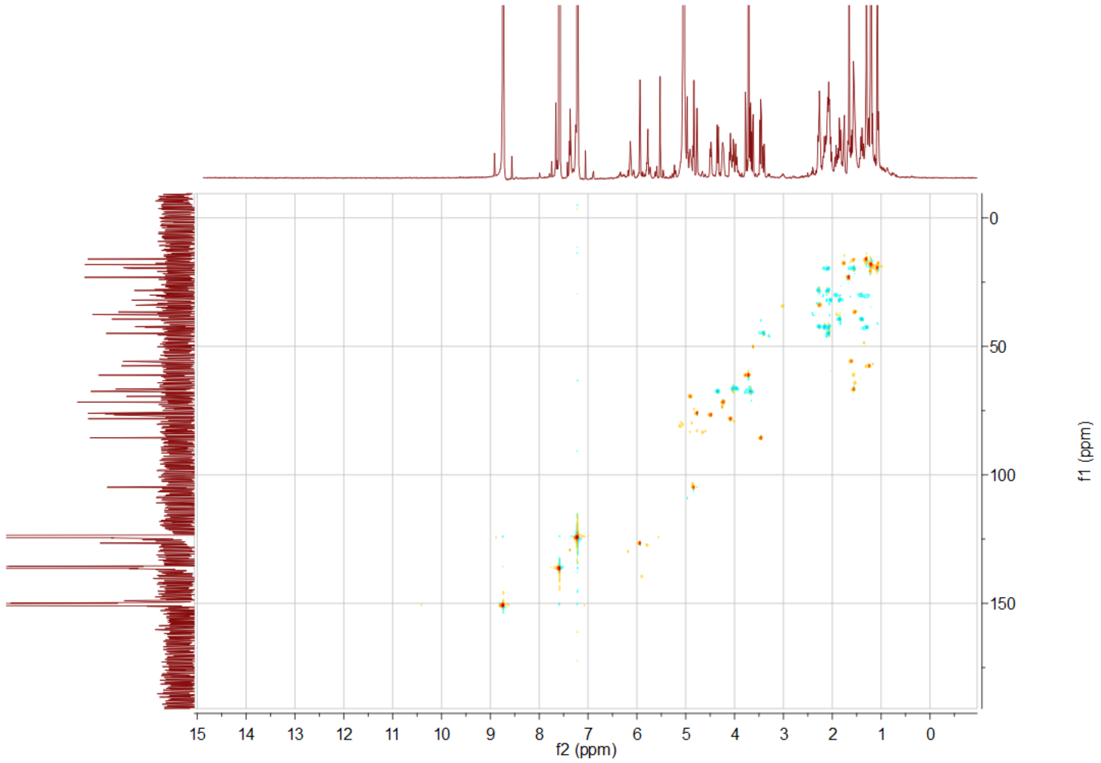

Figure S3. HSQC spectrum of Culcinoside A (**1**)

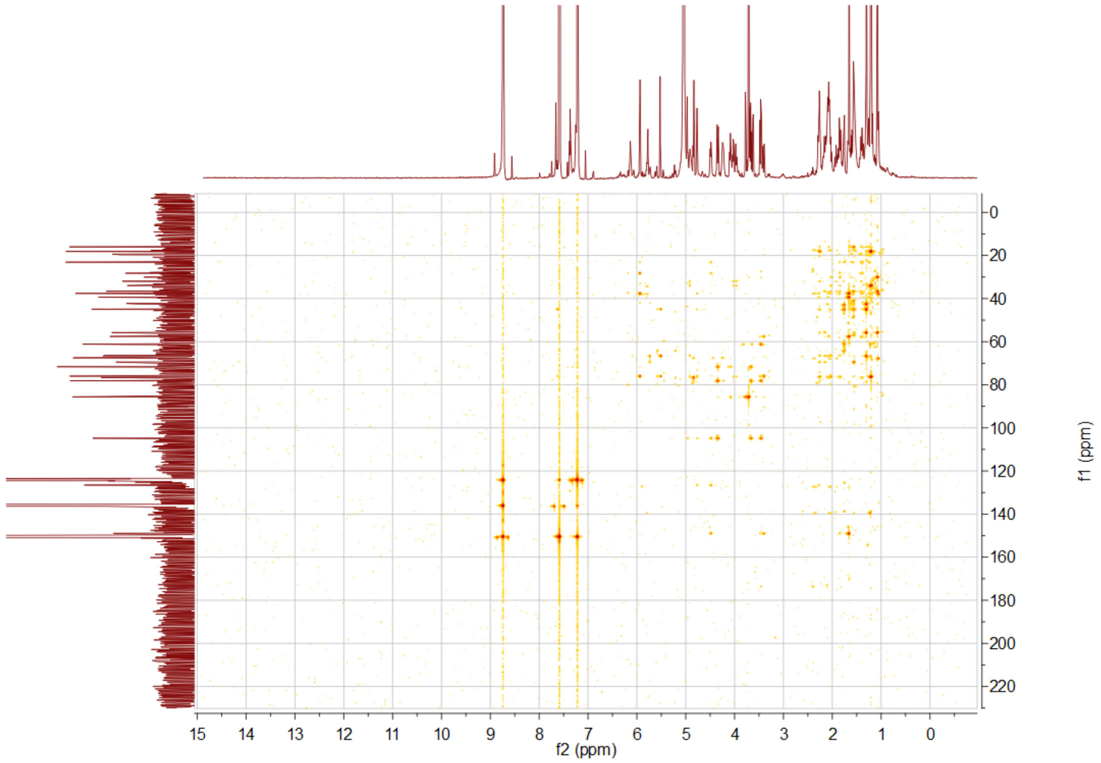

Figure S4. HMBC spectrum of Culcinoside A (**1**)

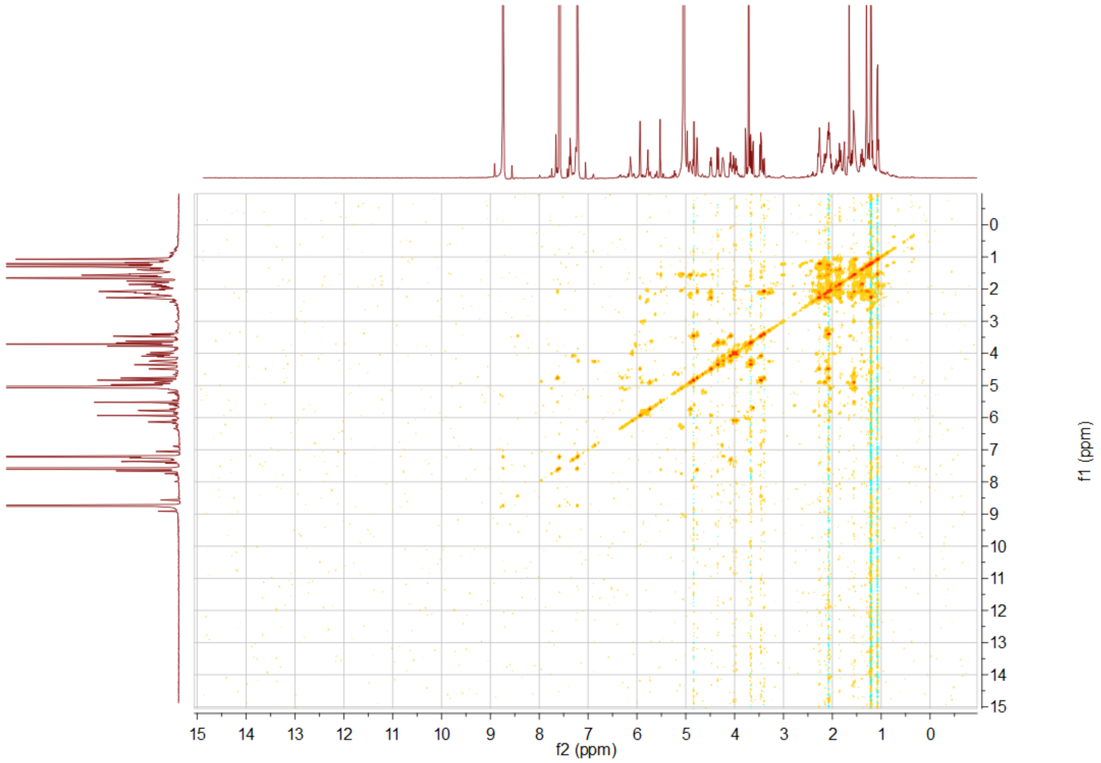

Figure S5. ^{1}H-^{1}H COSY spectrum of Culcinoside A (**1**)

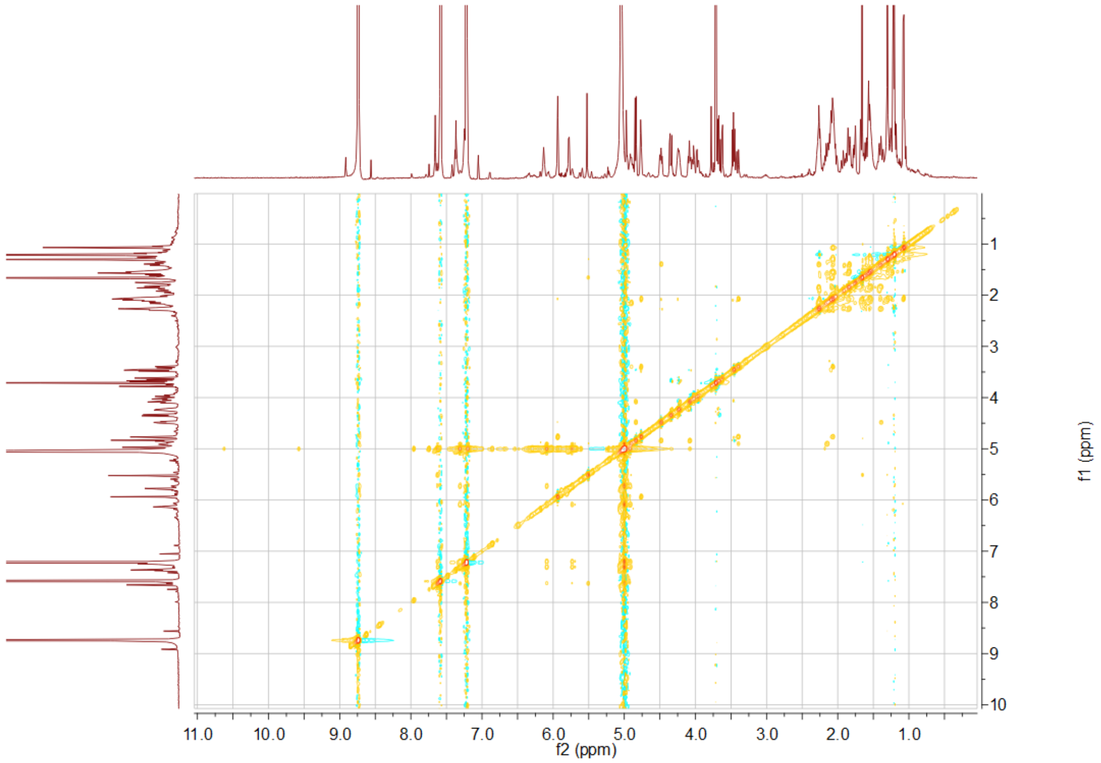

Figure S6. NOESY spectrum of Culcinoside A (**1**)

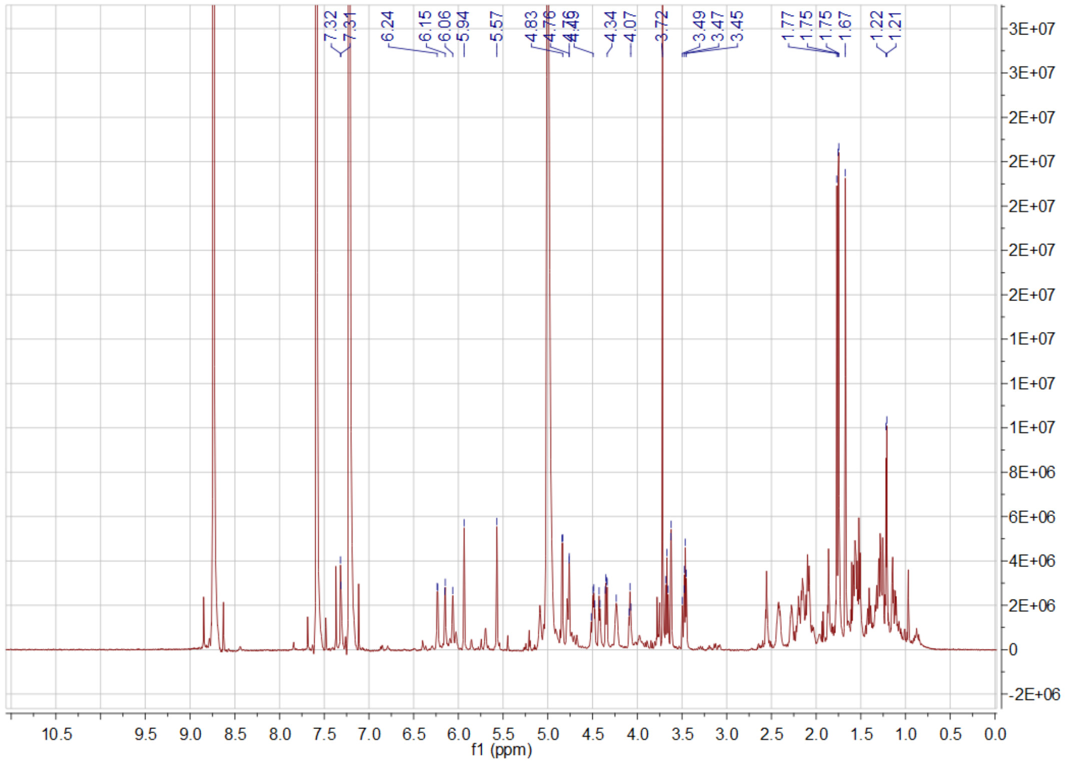

Figure S7. ^{1}H NMR (500 MHz, in C_5D_5N) spectrum of Culcinoside B (**2**)

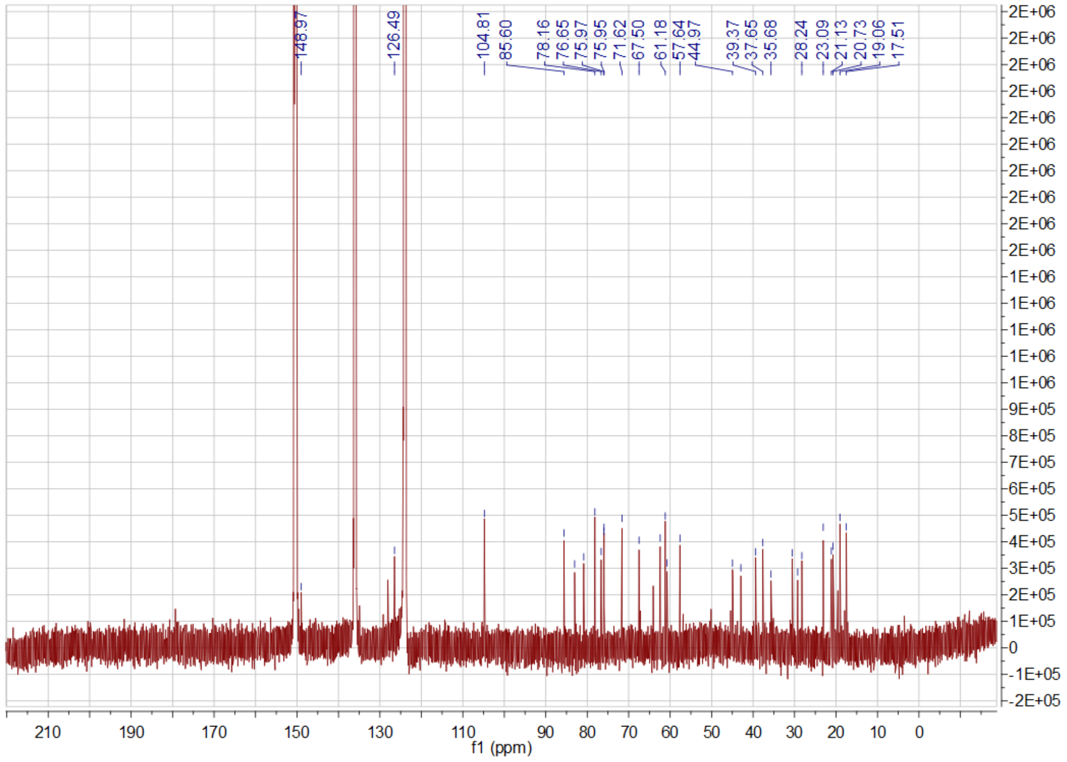

Figure S8. ^{13}C NMR (125 MHz, in C_5D_5N) spectrum of Culcinoside B (**2**)

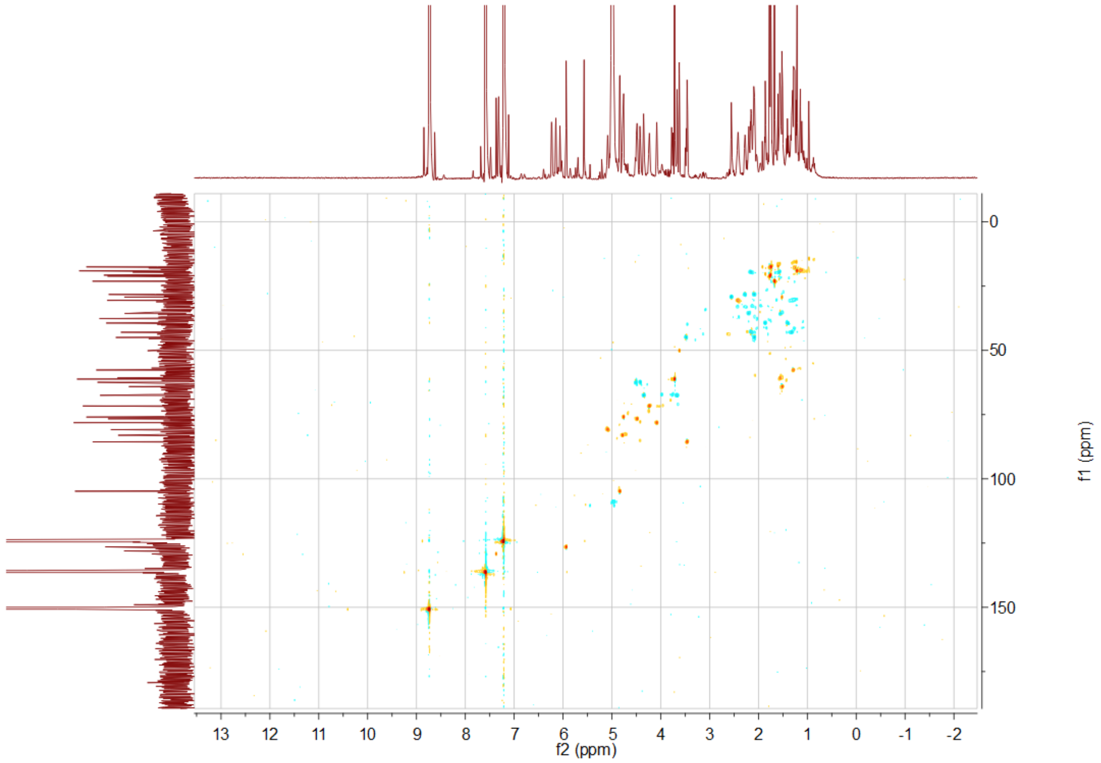

Figure S9. HSQC spectrum of Culcinoside B (**2**)

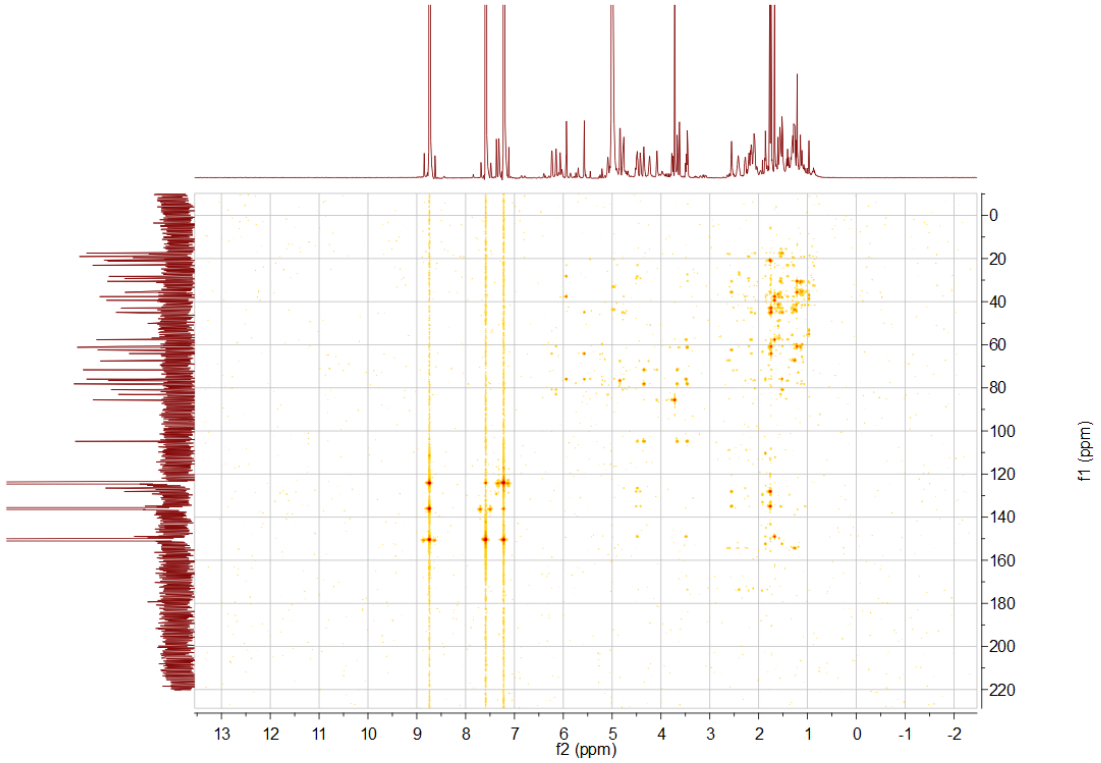

Figure S10. HMBC spectrum of Culcinoside B (**2**)

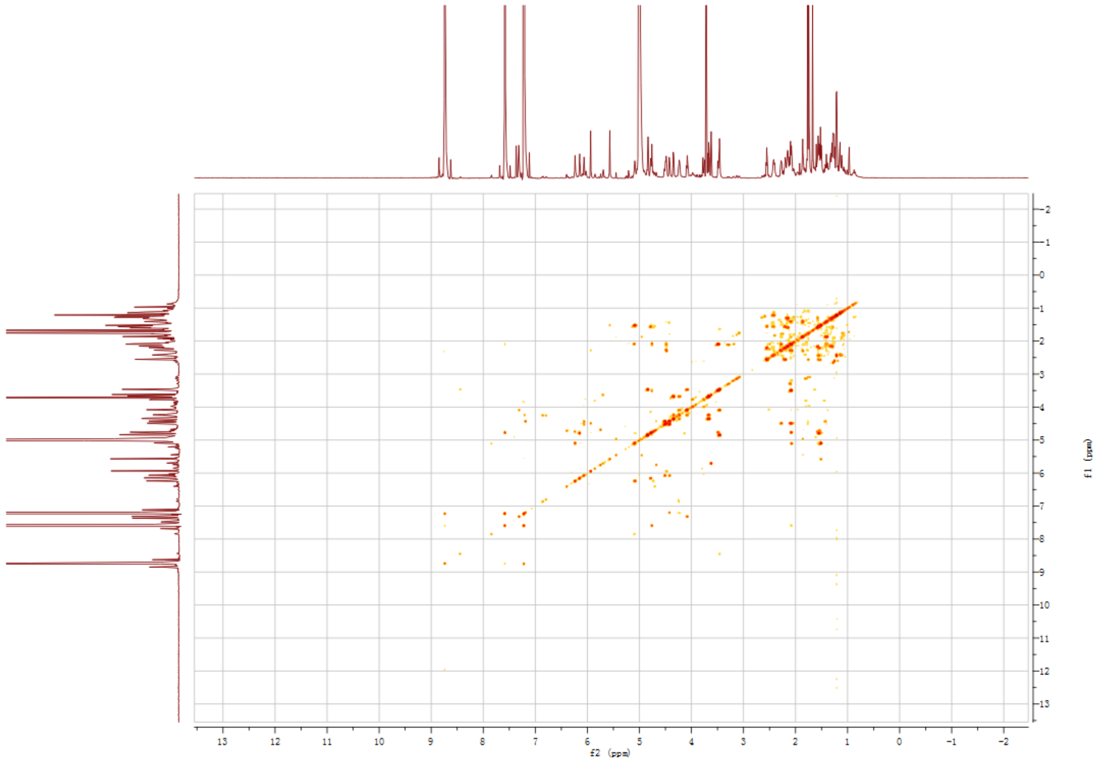

Figure S11. ^{1}H-^{1}H COSY spectrum of Culcinoside B (**2**)

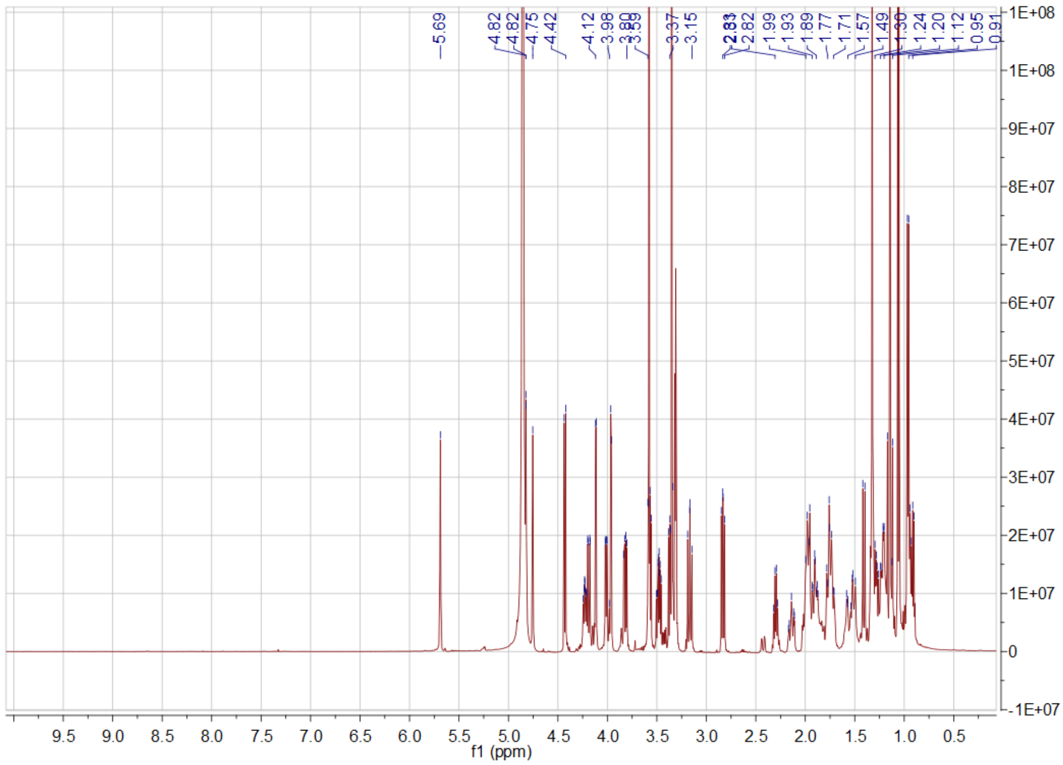

Figure S12. ^{1}H NMR (500 MHz, in CD_3OD) spectrum of Culcinoside C (**4**)

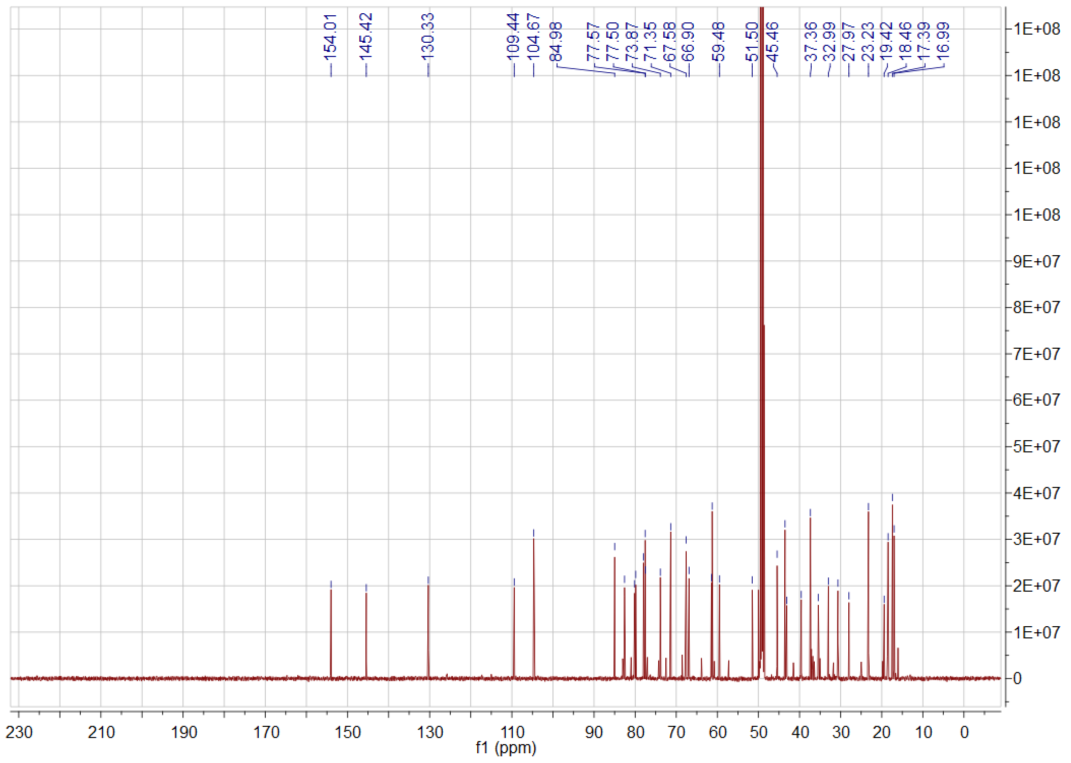

Figure S13. ^{13}C NMR (125 MHz, in CD_3OD) spectrum of Culcinoside C (**4**)

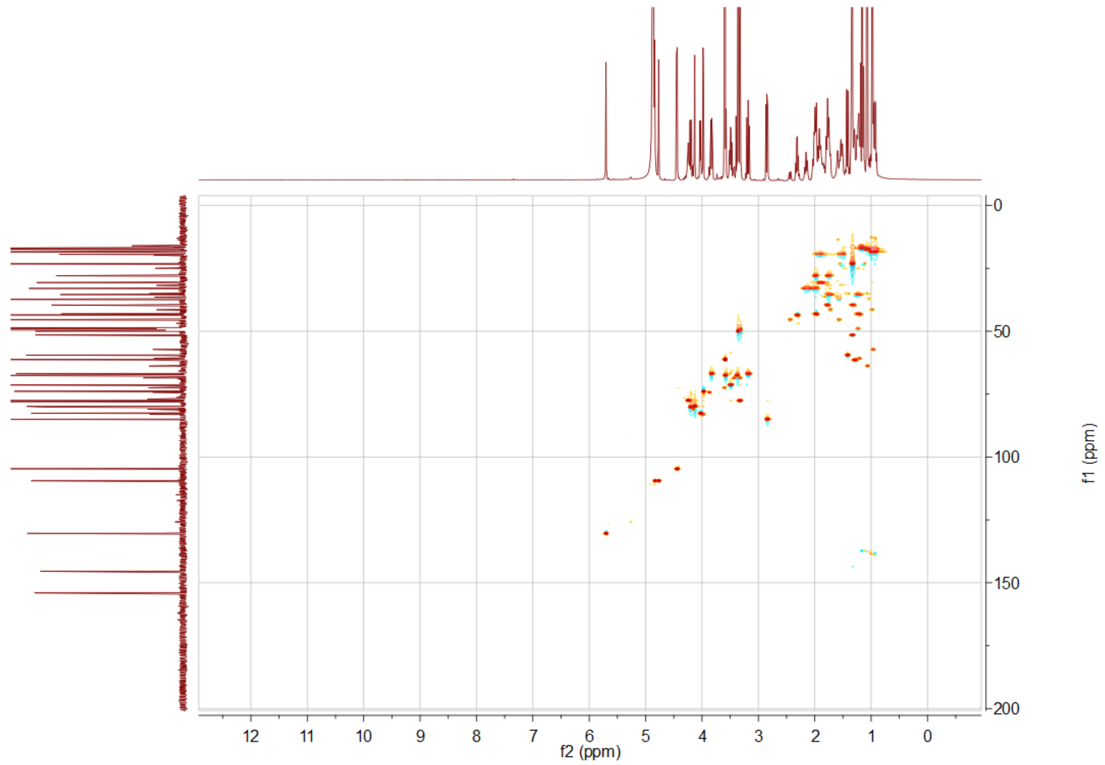

Figure S14. HSQC spectrum of Culcinoside C (**4**)

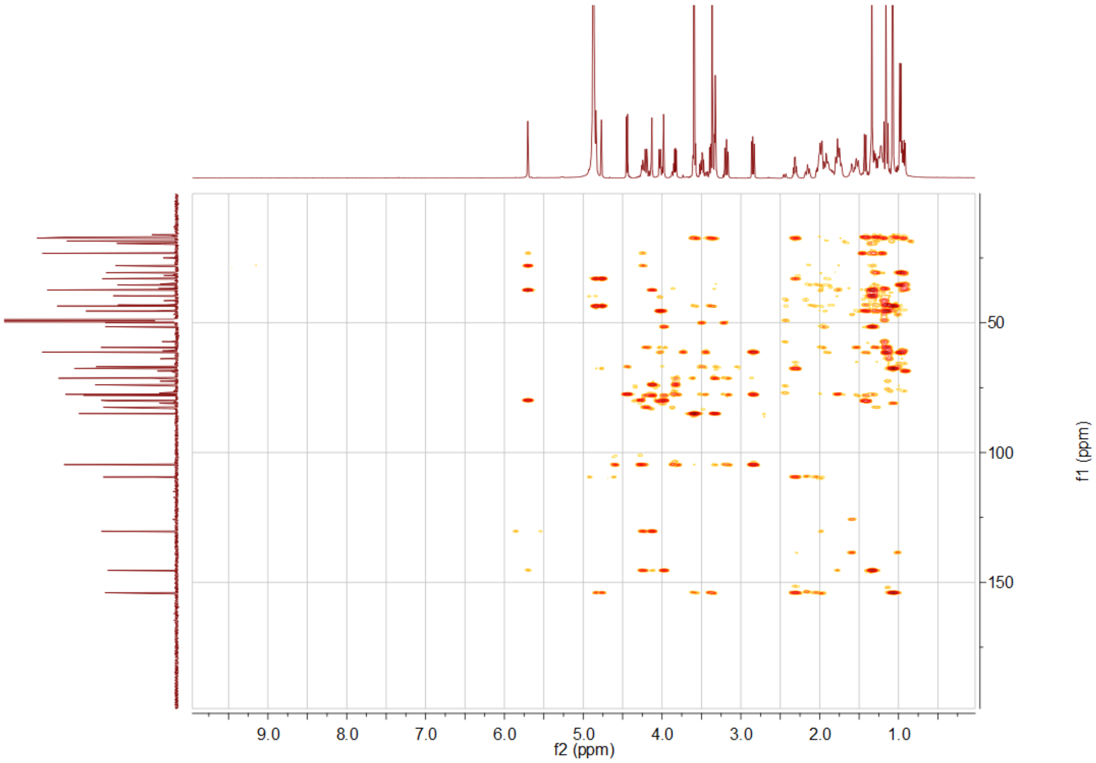

Figure S15. HMBC spectrum of Culcinoside C (**4**)

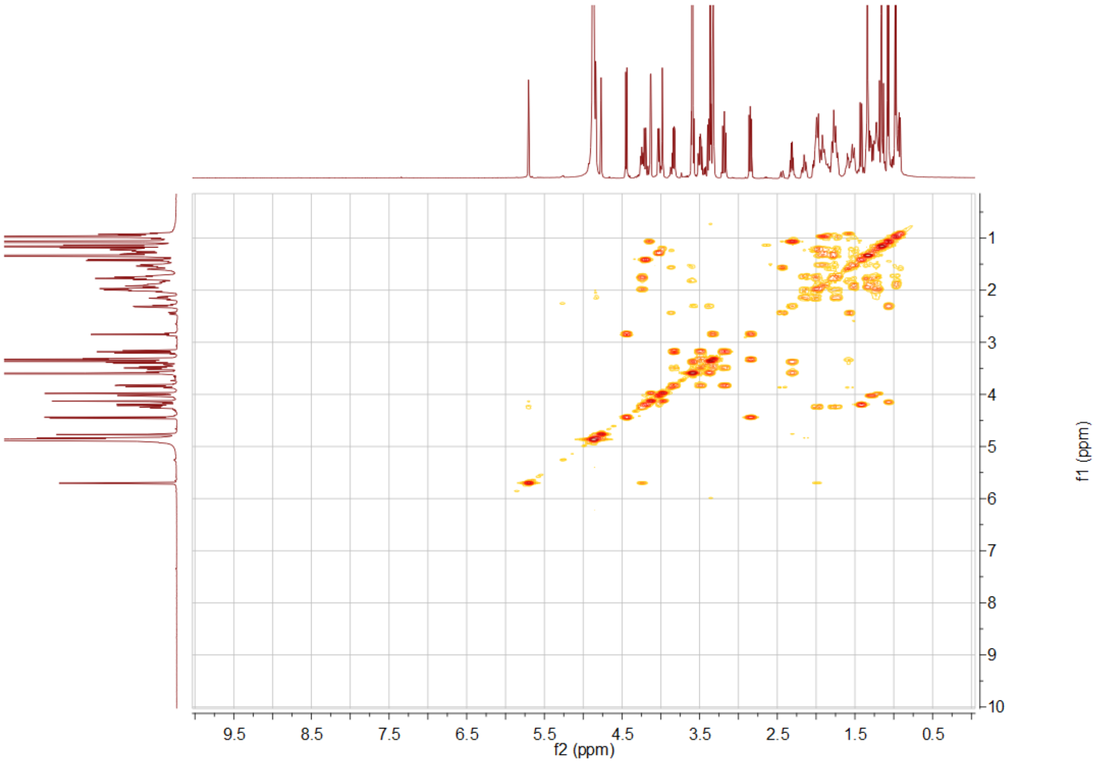

Figure S16. ^{1}H-^{1}H COSY spectrum of Culcinoside C (**4**)

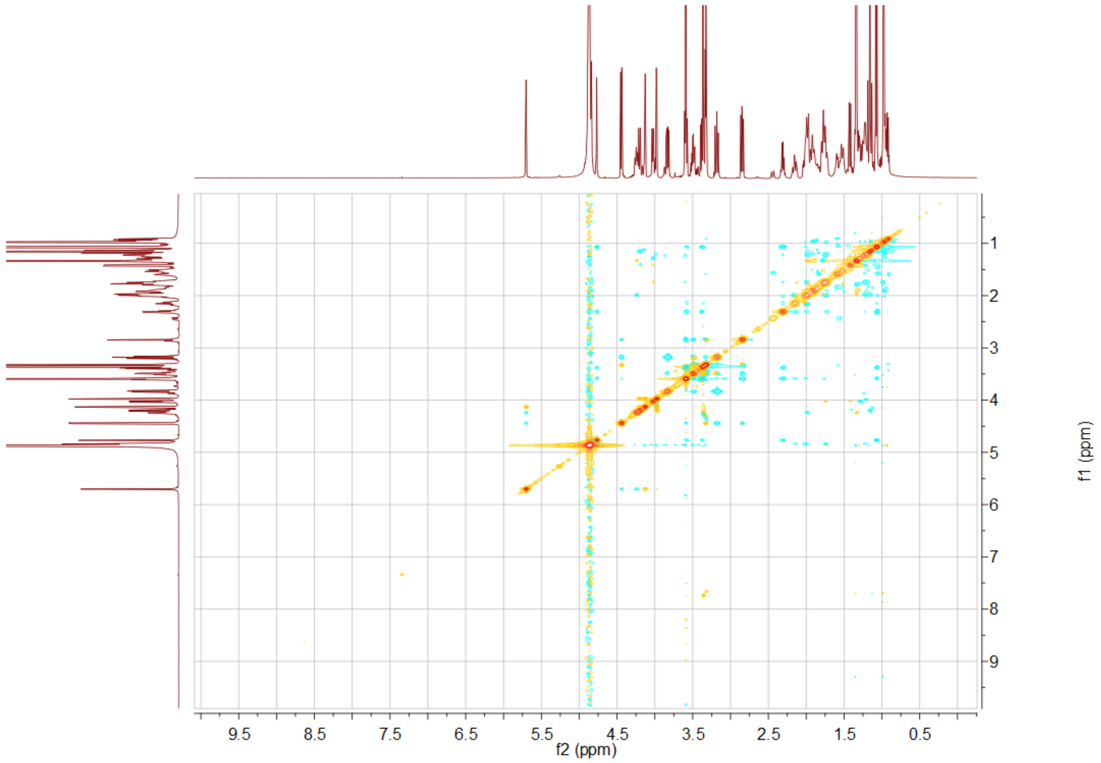

Figure S17. NOESY spectrum of Culcinoside C (**4**)

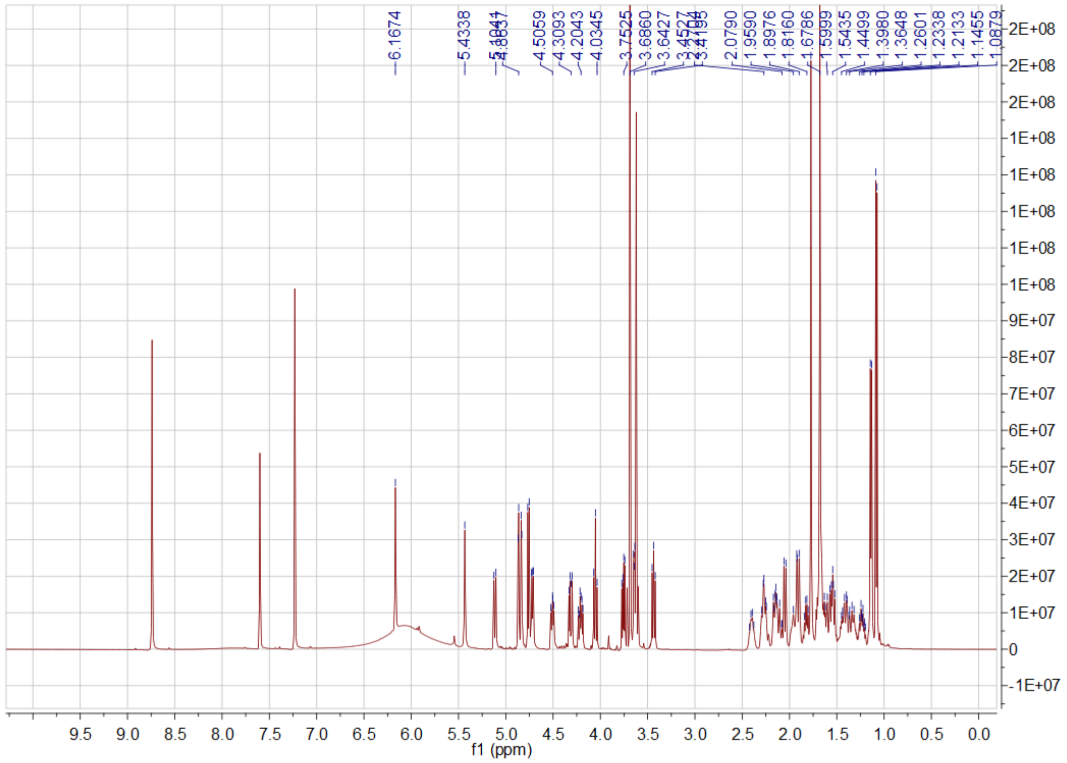

Figure S18. ^{1}H NMR (500 MHz, in C_5D_5N) spectrum of Culcinoside D (**7**)

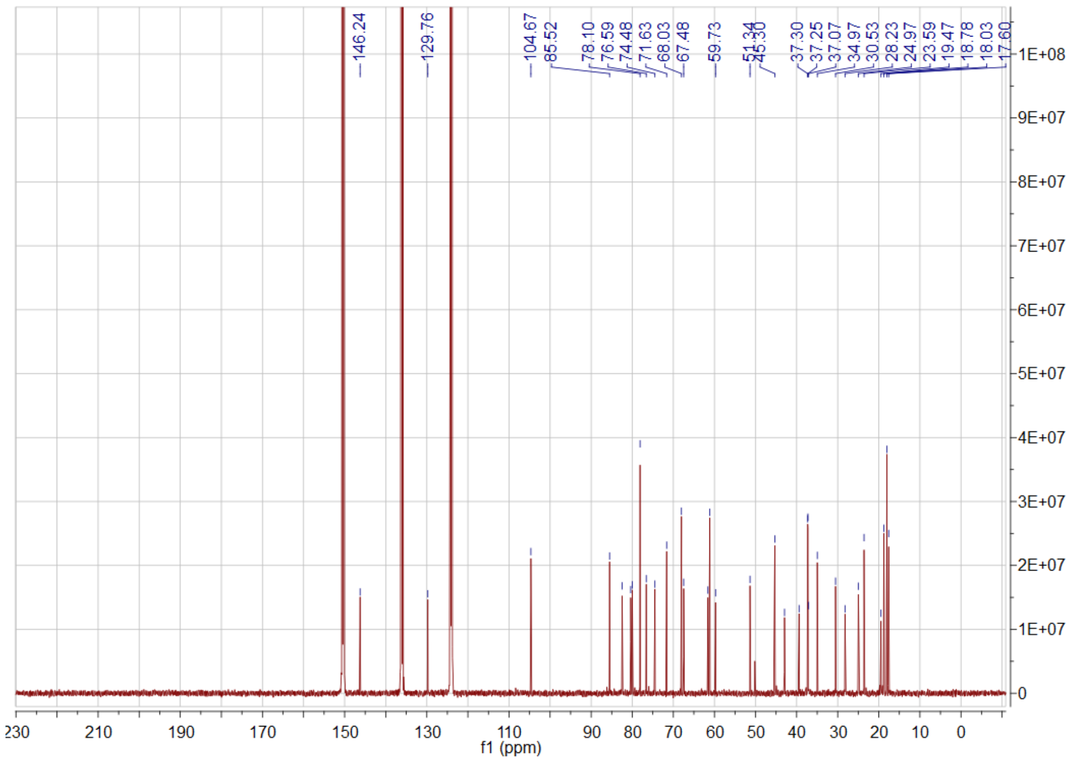

Figure S19. ^{13}C NMR (125 MHz, in C_5D_5N) spectrum of Culcinoside D (**7**)

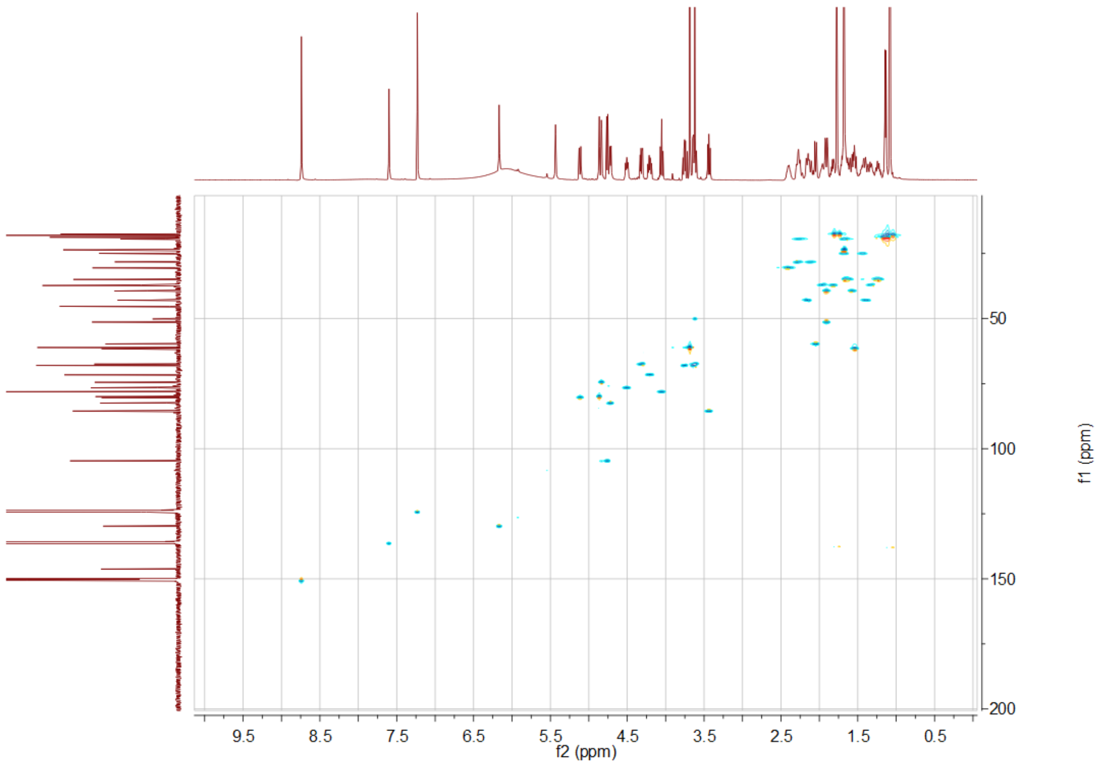

Figure S20. HSQC spectrum of Culcinoside D (**7**)

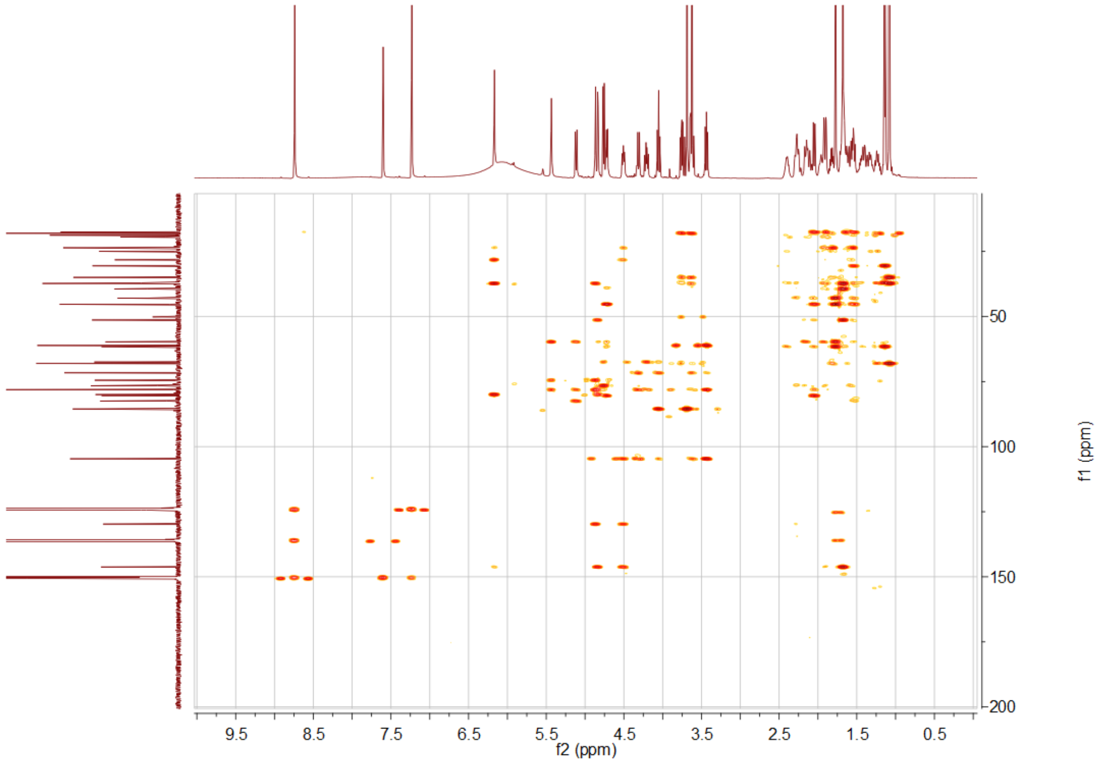

Figure S21. HMBC spectrum of Culcinoside D (**7**)

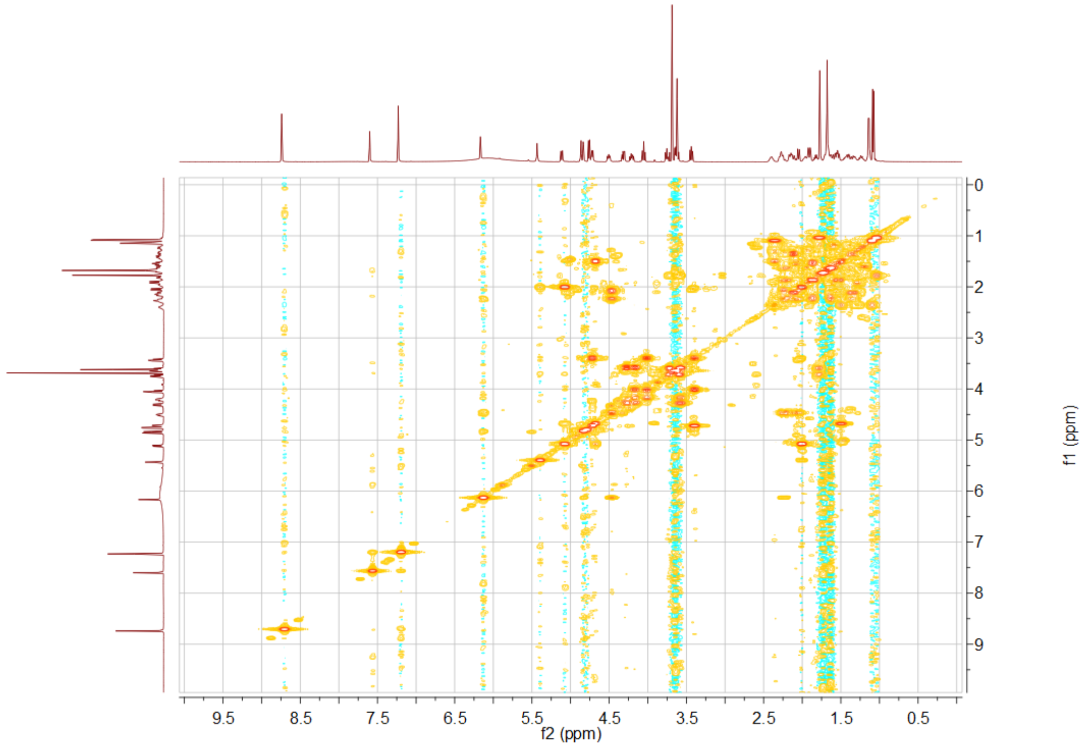

Figure S22. ^{1}H-^{1}H COSY spectrum of Culcinoside D (**7**)

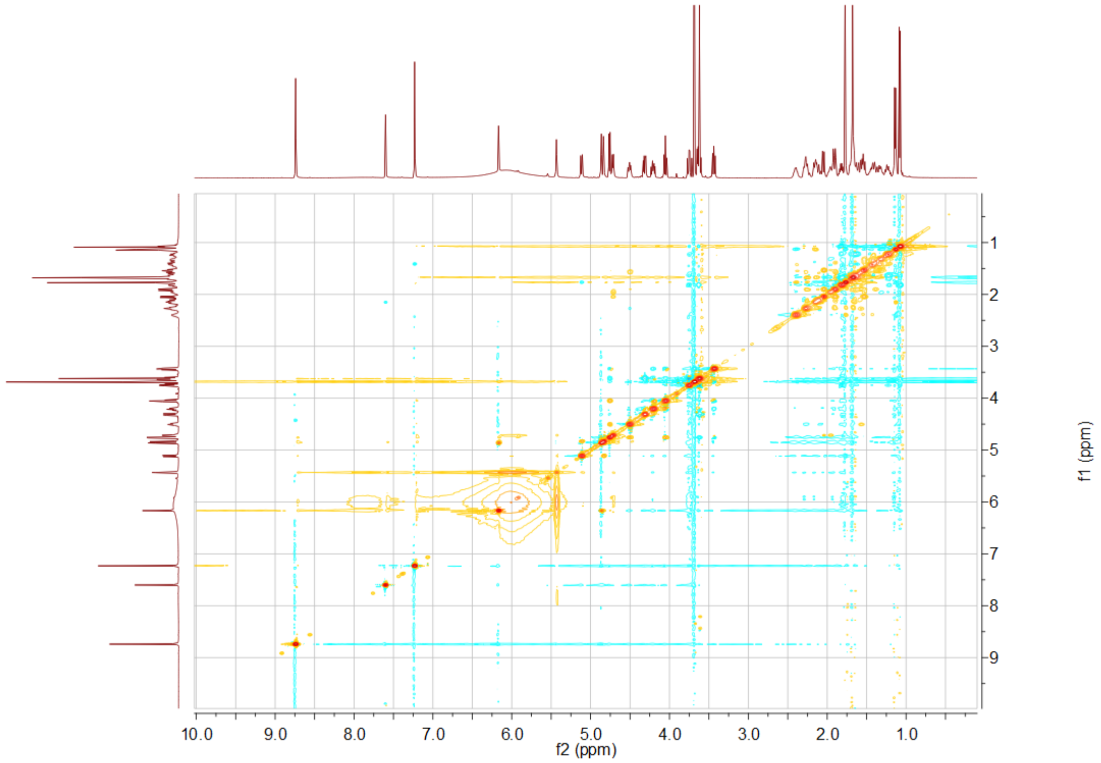

Figure S23. NOESY spectrum of Culcinoside D (**7**)

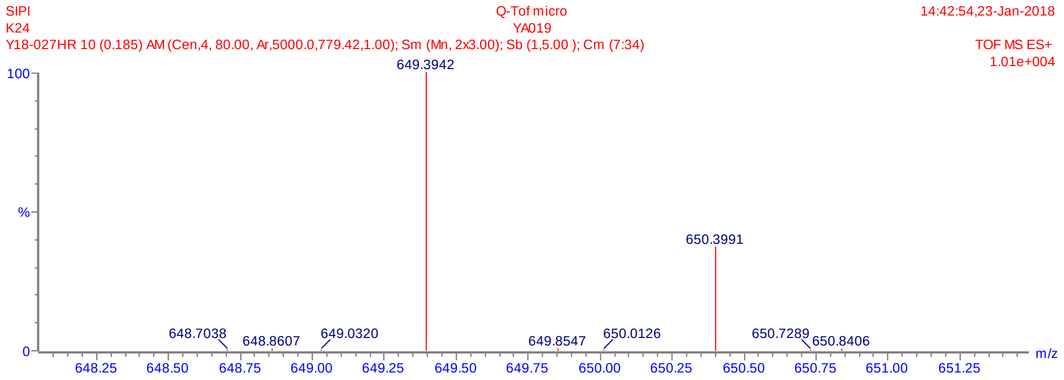

Figure S24.HRESIMS spectrum of Culcinoside A (**1**)

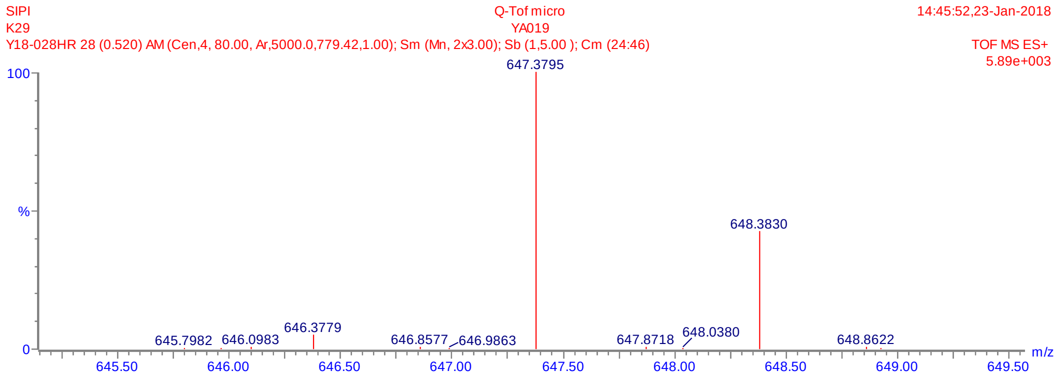

Figure S25. HRESIMS spectrum of Culcinoside B (**2**)

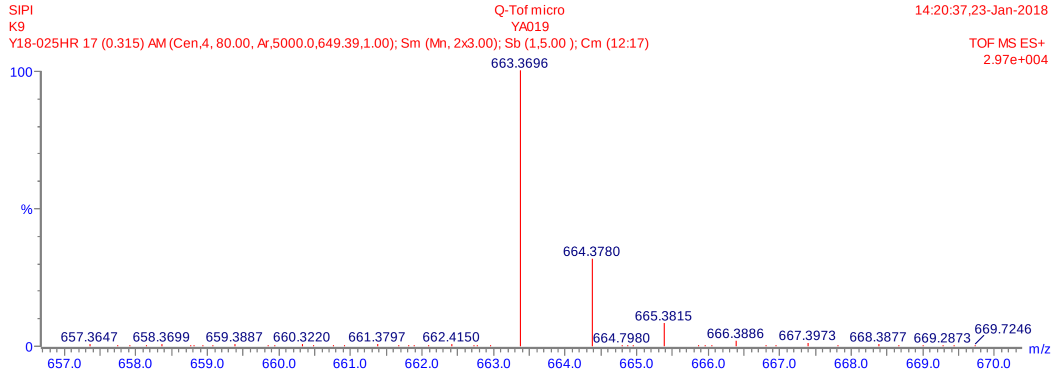

Figure S26.HRESIMS spectrum of Culcinoside C (**4**)

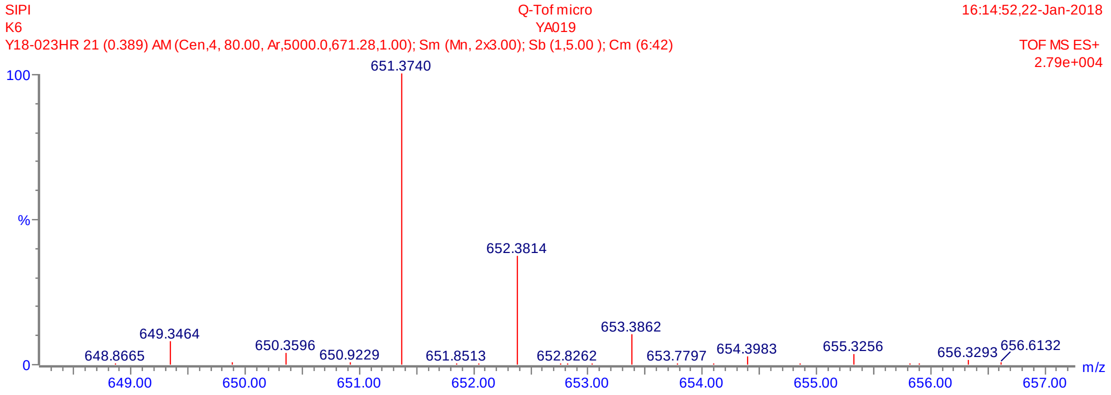

Figure S27.HRESIMS spectrum of Culcinoside D (**7**)
